# Physiological and proteome studies of maize (*Zea mays* L.) in response to leaf removal under high plant density

**DOI:** 10.1186/s12870-018-1607-8

**Published:** 2018-12-29

**Authors:** Shanshan Wei, Xiangyu Wang, Dong Jiang, Shuting Dong

**Affiliations:** 1 College of Agriculture/Key Laboratory of Crop Physiology, Ecology and Management, Ministry of Agriculture/Hi-Tech Key Laboratory of Information Agriculture of Jiangsu Province, Nanjing Agricultural University, Nanjing, 210095 Jiangsu Province People’s Republic of China; 2 State Key Laboratory of Crop Biology, College of Agriculture, Shandong Agricultural University, Tai’an, 271018 Shandong Province People’s Republic of China; 3 College of Life Science, Nanjing Agricultural University, Nanjing, 210095 Jiangsu Province People’s Republic of China

**Keywords:** Leaf removal, Light transmission rate, Maize, Photosynthesis, TMT label

## Abstract

**Background:**

Under high plant density, intensifying competition among individual plants led to overconsumption of energy and nutrients and resulted in an almost dark condition in the lower strata of the canopy, which suppressed the photosynthetic potential of the shaded leaves. Leaf removal could help to ameliorate this problem and increase crop yields. To reveal the mechanism of leaf removal in maize, tandem mass tags label-based quantitative analysis coupled with liquid chromatography–tandem mass spectrometry were used to capture the differential protein expression profiles of maize subjected to the removal of the two uppermost leaves (S_2_), the four uppermost leaves (S_4_), and with no leaf removal as control (S_0_).

**Results:**

Excising leaves strengthened the light transmission rate of the canopy and increased the content of malondialdehyde, whereas decreased the activities of superoxide dismutase and peroxidase. Two leaves removal increased the photosynthetic capacity of ear leaves and the grain yield significantly, whereas S_4_ decreased the yield markedly. Besides, 239 up-accumulated proteins and 99 down-accumulated proteins were identified between S_2_ and S_0_, which were strongly enriched into 30 and 23 functional groups; 71 increased proteins and 42 decreased proteins were identified between S_4_ and S_0_, which were strongly enriched into 22 and 23 functional groups, for increased and decreased proteins, respectively.

**Conclusions:**

Different defoliation levels had contrastive effects on maize. The canopy light transmission rate was strengthened and proteins related to photosynthetic electron-transfer reaction were up-regulated significantly for treatment S_2_, which improved the leaf photosynthetic capacity, and obtained a higher grain yield consequently. In contrast, S_4_ decreased the grain yield and increased the expressions of proteins and genes associated with fatty acid metabolism. Besides, both S_2_ and S_4_ exaggerated the defensive response of maize in physiological and proteomic level. Although further studies are required, the results in our study provide new insights to the further improvement in maize grain yield by leaf removal.

**Electronic supplementary material:**

The online version of this article (10.1186/s12870-018-1607-8) contains supplementary material, which is available to authorized users.

## Background

Maize (*Zea mays* L.) yield has advanced through breeding complemented with evolving management technologies [1]. Increasing the maize plant population is an effective practice that has undergone a constant evolution over the years [2]. Under high plant densities, however, intensifying competition occurred among individual plants, and led to overconsumption of energy and nutrients including stronger root systems or bigger leaf area [3]. Meanwhile, the close distances between plants in the group resulted in an almost dark condition in the lower strata of the canopy, which suppressed the photosynthetic potential of the leaves [4, 5]. Nevertheless, leaves in the middle canopy are the main source of corn grain yield, and the photosynthetic intensity is closely related to yield production [6, 7]. Besides, shading condition could accelerate the reduction of chlorophyll (Chl) content and leaf area of leaves at lower canopy status [8].

Excising vegetative organs partially is an effective method to modify the canopy structure, which is benefit to improve the light environment within the canopy, and ultimately alter crop yield [9–11]. Nevertheless, the response of yield to leaf removal levels differs greatly [12]. When plants are injured after artificial defoliation, eaten by animals or pests, the leaf area decrease thereafter [13, 14], however residual organs have a compensating effect when the photosynthetic organs injured above a certain threshold level [15, 16]. The effect (negative, positive, or zero) of source-reducing on plants growth depends on the frequency and intensity of defoliation [17]. Liu et al. [12] has demonstrated that defoliation above the cob decreased leaf area index significantly, whereas it markedly improved light condition within the canopy. Besides, removing the uppermost two or four leaves in maize appeared to stimulate an increase in net photosynthetic rate (P_n_), stomatal conductance, and Chl content of the ear leaf. Hao et al. [18] also evidenced that an increase in P_n_ of the remaining ear leaf came up by excising 1/4 and 1/2 of maize leaves over the whole plant. Increased intensity of leaf removal, however, do not conduce to maintain the photosynthetic ability of remaining leaves during late filling stage [19]. The photosynthesis extent of leaves during grain filling can be affected by canopy structure [20] and the corresponding variations in light conditions may lead to changes in the expression levels of proteins, which invariably leads to changes in plant metabolism [21].

Leaf removal has also been reported affecting antioxidant metabolism of plants [22], for instance, altered the activities of superoxide dismutase and peroxidase as well as the content of malondialdehyde [19, 23]. To date, though several physiology variations induced by leaf removal have been studied in maize and other plants, there is still little published information at the proteomic level regarding the effects of leaf removal on maize characteristics under high plant density. Therefore, we employed a quantitative proteomic analysis based on tandem mass tag (TMT) labels, coupled with liquid chromatography-tandem mass spectrometry (LC-MS/MS), to capture the differential protein expression profiles of maize subjected to defoliation. This research compared changes in physiology and proteins induced by different leaf removal treatments using a high-yield and density-tolerance variety under a optimized density, hoping to elucidate the physiological mechanism of leaf removal on maize production and provide a theoretical basis for further improvement in maize grain yield.

## Methods

### Experimental design

The experiment was conducted at the Corn Research Center of Shandong Agricultural University, Tai’an, Shandong Province, China (36° 10′ N, 117° 09′ E) from June 18 to October 8 during 2015 growing season. This area has a semi-humid, warm temperate continental climate with monsoons. The average content of organic matter in the tillage layer was 18.6 g kg^− 1^ and the total nitrogen (N), rapidly available phosphorous (P), and rapidly available potassium (K) were 1.03 g kg^− 1^, 43.05 mg kg^− 1^ and 78.91 mg kg^− 1^, respectively.

The summer maize hybrid Denghai 618 (a high-yield and density-tolerance variety grown extensively in North China) was selected as the material for testing. Maize seeds were planted with hand planters at a uniform density of 9.75 plants m^− 2^, which was a optimum density for Denghai 618 selected during 2013 to 2014 growing seasons (a relatively high density for the growing conditions of the North China Plain). Pre-sowing, phosphorus (P_2_O_5_) and potassium (K_2_O) fertilizer were applied at a rate of 90 kg·ha^− 1^ and 120 kg ha^− 1^ per plot, respectively. Urea (N 46%) was applied by furrow at six and twelve leaves unfolded stage respectively, at a rate a 180 kg ha^− 1^ each time.

Three treatments were set up in our study, including the uppermost two leaves removal (S_2_), the uppermost four leaves removal (S_4_) and the control with no leaf removal (S_0_). Plants were grown until silking stage, when leaf removal treatments were imposed. Each treatment had three replicate plots, with each plot area measuring 3 m × 15 m, and the spacing between rows was 0.6 m. Besides, irrigation, weeds, diseases and insect pests were controlled adequately during the whole growing season so that no factors other than leaf removal affect plants’ growth.

### Sampling

For plant sampling, the uniform and healthy maize plants were marked at silking stage. The middle portion of five marked ear leaves from five individual plant of each plot was collected and mixed as one replicate at three days and seven days after leaf removal and plunged directly into liquid nitrogen, then stored at − 80 °C prior to analysis. The remaining marked plants were used to determine photosynthetic parameters. At physiological maturity, 20 ears from three center rows of each plot were harvest to measure yield (adjusted to a moisture content of 15.5%), kernel number per ear (KN) and 1000-kernel weight (TKW). Harvest index (HI) was calculated as the ratio of grain yield to the total above-ground biomass.

### Physiological measurements

The plant canopy digital image analyzer (CI-100, CID Bio-Science, Inc. USA) was used to calculate the light transmission rate, and the hemispheric gray images of ear and bottom layers were also taken. In each plot, the photosynthetic effective radiation (PAR) at the top, the ear and bottom (four leaves below the ear leaf) layers were taken. The PAR values at ear and bottom layers for each plot were the average of five measurements. Light transmission rate (%) was calculated as the following equation.

Light transmission rate (%) = PAR of the determined layer (ear or bottom) / PAR of the top canopy layer × 100%.

Gas exchange parameters, which including net photosynthetic rate (P_n_), stomatal conductance (g_s_) and intercellular CO_2_ concentration (C_i_), were measured using a portable photosynthesis system (CIRAS-II, UK). The artificial light was set at 1600 μmol m^− 2^ s^− 1^ and CO_2_ concentration in the leaf chamber was maintained at 360 μmol mol^− 1^ using a CO_2_ injector. The measurements were conducted between 09: 00 AM and 11: 00 AM and each treatment had three replications.

Three representative plants were selected to determine the green leaf area (GLA) nondestructively and leaf area index (LAI) was then calculated. The equations were as GLA = ∑ (leaf length × maximum width × 0.75); LAI = GLA × n / S, where n is the number of plants within a unit area of land and S is the unit area of land.

Leaf chlorophyll content was determined using spectrometry, following standard methods [24]. Nitroblue tetrazolium and guaiacol colorimetry methods [25] were used to measure the activities of superoxide dismutase (SOD) and peroxidase (POD), respectively. The content of malondialdehyde (MDA) was measured with thio-barbituric acid method [26].

### TMT-based quantitative proteomics analysis

Samples collected at three days after leaf removal were used for the TMT-based proteomics analysis (3 biological replicates × 3 treatments). Total proteins from each sample were extracted using the trichloroacetic acid (TCA)-acetone precipitation method. First, the samples were ground in liquid nitrogen and transferred to 5-mL centrifuge tubes. Then, lysis buffer (8 M urea, 1% Triton-100, 65 mM DTT and 0.1% Protease Inhibitor Cocktail) was added to the tubes, which were sonicated three times on ice using a high-intensity ultrasonic processor (Scientz). Next, the remaining debris was removed by centrifugation at 20,000 g at 4 °C for 10 min. Finally, the protein was precipitated with cold 15% TCA for 2 h at − 20 °C. The supernatant was discarded after centrifuging at 4 °C for 10 min. The remaining precipitate was washed with cold acetone three times. The protein was resuspended in buffer (8 M urea, 100 mM TEAB, pH 8.0), and the protein concentration was determined with a 2-D Quant kit according to the manufacturer’s instructions. Then, trypsin digestion was carried out with 10 mM DTT for 1 h at 37 °C, and 20 mM IAA was added to alkylate the proteins for 45 min at room temperature in the dark. This protein sample was diluted by adding 100 mM TEAB to a urea concentration of less than 2 M. Finally, trypsin was added in a 1:50 *w*/w ratio of trypsin to protein for a first overnight digestion and 1: 100 w/w ratio of trypsin to protein for a second 4-h digestion. Approximately 100 μg protein for each sample was digested with trypsin for the following experiments.

After trypsin digestion, six-plex TMT labelling (Thermo Scientific) was performed following the manufacturer’s protocol. Briefly, one unit of TMT reagent (defined as the amount of reagent required to label 50 μg of protein) was thawed and reconstituted in 24 μL ACN. The peptide was reconstituted in 0.2 M TEAB, mixed with the TMT reagent, and incubated for 2 h at room temperature. The samples were desalted in a Strata X C18 SPE column (Phenomenex) and vacuum-dried. Each dried peptide sample was fractionated using high-pH reverse-phase HPLC with an Agilent 300 Extend C18 column (5-μm particles, 4.6-mm ID, 250-mm length). Eighteen fractions were collected.

The peptides were dissolved in 0.1% formic acid (FA) and loaded directly onto a reversed-phase pre-column (Acclaim PepMap 100, Thermo Scientific). The peptides were separated using a reversed-phase analytical column (Acclaim PepMap RSLC, Thermo Scientific). The peptide samples were subsequently eluted with a four-step linear gradient of solvent B (0.1% FA in 98% ACN): 6–22%, 26 min; 22–35%, 8 min; 80%, 3 min; and 80%, hold, 5 min. A constant flow rate was maintained as 300 mL/min with an EASY-nLC 1000 ultra-performance liquid chromatography (UPLC) system. The resulting peptides were processed using a Q Exactive™ Plus hybrid quadrupole-Orbitrap mass spectrometer (Thermo Fisher Scientific) coupled online to the UPLC. The MS was processed with a data-dependent procedure that alternated between single-MS and MS/MS scans. Intact peptides were detected in the Orbitrap (350–1800 m/z, 70,000 resolution) and subjected to 20 MS/MS scans using an NCE setting of 31. The top 20 precursor ions above a threshold ion count of 1E4 in the MS survey scan were identified with 30.0-s dynamic exclusion. Ion fragments were detected in the Orbitrap at 17,500 resolution. Automatic gain control (AGC) was set as 5E4 ions to prevent overfilling of the ion trap.

The Mascot search engine (v.2.3.0) was used to search the resulting MS/MS data against the UniProt *Zea mays* database (58,493 sequences). The cleavage enzyme was specified as trypsin/P, and two missing cleavages were allowable. The mass error was set to 10 ppm for precursor ions and to 0.02 Da for fragment ions. Carbamidomethyl on Cys, TMT-6plex (N-term), and TMT-6plex (K) were specified as fixed modifications, and oxidation of Met was specified as a variable modification. FDR was adjusted to ≤1%, and the peptide ion score was set at ≥20.

### Bioinformatics analysis

The UniProt-GOA database (http://www.ebi.ac.uk/GOA) was used to obtain the Gene Ontology (GO) proteome annotation. First, the identified protein ID was converted to a UniProt ID, and then these were mapped to GO IDs using the protein ID. If some identified proteins were not annotated by the UniProt-GOA database, InterProScan was used to annotate the GO function of the protein based on a protein sequence-alignment method. Then, the proteins were classified into three categories using the GO annotation: biological process, cellular component, and molecular function. For each category, a two-tailed Fisher’s exact test was used to test the enrichment of the differentially expressed protein against all identified proteins. Correction for multiple hypothesis testing was carried out using standard false discovery rate control methods. A GO with a corrected *P*-value ≤0.05 was considered significant.

### Quantitative real-time polymerase chain reaction (qRT-PCR)

The analysis of qRT- PCR was performed following the method of Wang et al. [27]. Total RNA was extracted from 0.05–0.1 g maize ear leaf by the use of RNAiso Plus reagent (Takara Bio, Japan). The cDNA were synthesized by using HiScript II Q RT SuperMix for qPCR (+gDNA wiper) (Vazyme Bio, China). Specific primers for each gene tested in our study are listed in Additional file 1: Table S1. The relative expression level of each gene was calculated according to the 2^−ΔΔCt^ method, using *Zmactin* as the reference gene. The equation is:

$$ \varDelta \varDelta {C}_t=\left({C}_t\ \mathrm{target}\ \mathrm{gene}-{C}_t\ \mathrm{reference}\ \mathrm{gene}\right){S}_2/{S}_4-\left({C}_t\ \mathrm{target}\ \mathrm{gene}-{C}_t\ \mathrm{reference}\ \mathrm{gene}\right){S}_0 $$

### Statistical analysis

SPSS 18.0 (SPSS Institute Inc.) was used to perform analyses of variation (ANOVAs) for physiological parameter. The results for each parameter are presented as the means of the three replicates (except for kernel numbers). Differences were judged by the least significant differences (LSD) test, and the significance level was set at the 0.05 probability level. Figures were plotted using SigmaPlot 12.0 (Systat Software Inc.).

## Results

### Yield and physiological indices

The grain yield and yield components were different between leaf removal treatments and the control (Table 1). Relative to S_0_, S_2_ plants obtained significantly greater (*P* ≤ 0.05) 1000-kernel weight, total dry matter and harvest index, which resulted in an increase in final yield of 5.2%. In contrast, S_4_ plants had significantly lower (*P* ≤ 0.05) kernel numbers, 1000-kernel weight, total dry matter and harvest index, which resulted in a decrease in final yield of 11%.

**Table 1:** Effect of leaf removal on grain yield (15.5 g kg^− 1^ water content) and yield components

Treatment	Grain yield	Kernel Numbers	1000-Kernel weight	Total dry matter	Harvest index
	(Mg ha^−1^)	(no. ear^−1^)	(g)	(Mg ha^− 1^)	(%)
S_0_	15.4 ± 0.2b	457.6 ± 33.8a	306.5 ± 3.4b	25.6 ± 0.4b	50.9 ± 0.49b
S_2_	16.2 ± 0.1a	456.1 ± 25.3a	317.3 ± 1.9a	26.6 ± 0.2a	51.5 ± 0.27a
S_4_	13.7 ± 0.1c	424.5 ± 15.1b	290.2 ± 1.6c	23.4 ± 0.7c	49.6 ± 0.39c

Note: Data are means ± SE (n = 3, except for kernel number *n* = 20). Different letters means within a column mean significant differences at 5%

**Fig. 1:**
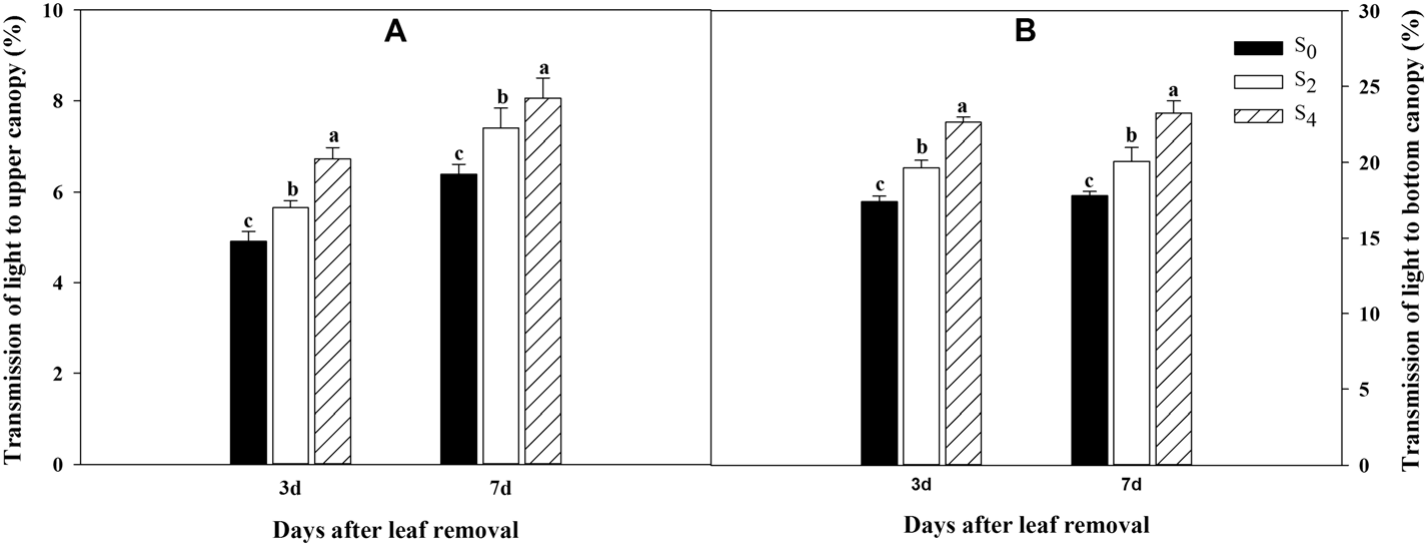
Canopy light transmission at the bottom canopy (**a**) and the middle canopy (**b**) in response to different levels of leaf removal at three and seven days after defoliation. S_0_ refers to control (no leaf removal); S_2_ and S_4_ refer to the removal of two or four leaves, respectively, from top of the plant. Bars indicate ± standard error of the mean (*n* = 3). Different small letters in each group indicate significant differences at *P* ≤ 0.05

**Fig. 2:**
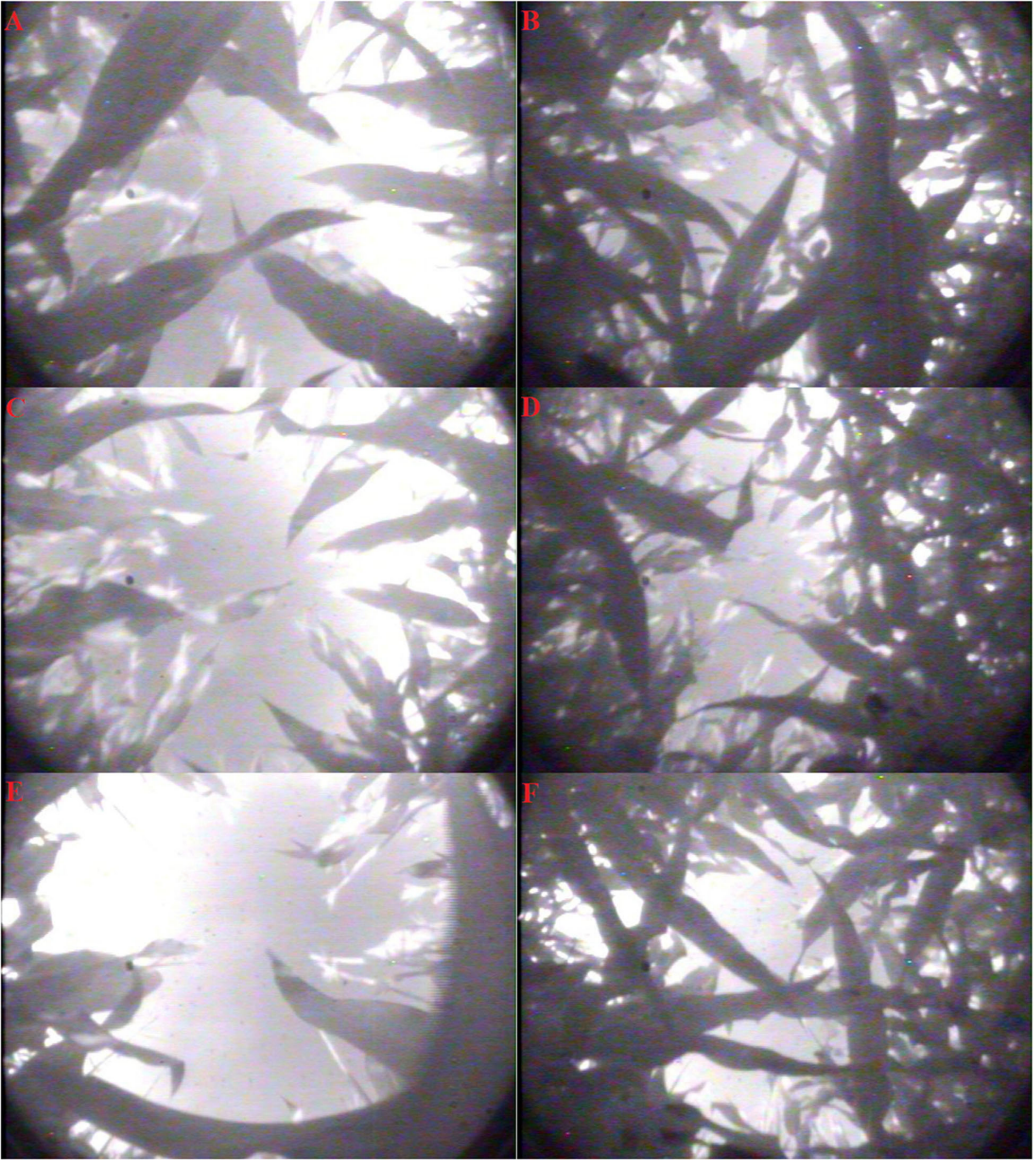
The hemispheric gray images within the maize canopy on the day of defoliation. **a**, **c**, and **e** represent hemispheric gray images of ear layer for control (no leaf removal, S_0_), two leaves removal (S_2_), and four leaves removal (S_4_), respectively; **b**, **d** and **f** represent hemispheric gray images of bottom layer (four leaves below the ear leaf) for S_0_, S_2_, and S_4_, respectively

Leaf area index (LAI) were decreased significantly after leaf removal (*P* ≤ 0.05, Table 2). Compared to S_0_, the LAI after defoliation in S_2_ and S_4_ decreased for 5.8 and 19.5%, respectively. Light transmission rate of canopy was significantly enhanced (*P* ≤ 0.05) at the level of the ear leaf strata and the bottom leaf strata with the increased levels of leaf removal (Fig. 1). In addition, by watching the hemispheric gray images (Fig. 2), we also intuitively found that there was an increased light transmittance, especially in the ear layer, induced by leaf removal.

**Table 2:** Effect of leaf removal on leaf area index (LAI) and activities of superoxide dismutase (SOD), peroxidase (POD) and malondialdehyde (MDA)

Treatment	LAI	SOD (U g^−1^ FW min^− 1^)		POD (U g^− 1^ FW min^− 1^)		MDA (μmol g^− 1^ FW)	
	0 d	3 d	7 d	3 d	7 d	3 d	7 d
S_0_	6.23 ± 0.09 a	494.8 ± 3.6 a	473.4 ± 3.9 a	86.4 ± 0.2 a	74.9 ± 1.1 a	15.4 ± 0.1 c	24.3 ± 0.7 c
S_2_	5.87 ± 0.09 b	486.4 ± 1.4 b	461.0 ± 4.5 b	84.9 ± 0.5 b	72.9 ± 0.2 b	16.0 ± 0.2 b	26.0 ± 0.2 b
S_4_	5.02 ± 0.08 c	478.3 ± 2.3 c	446.7 ± 4.9 c	82.6 ± 0.6 c	70.6 ± 0.2 c	16.3 ± 0.2 a	27.5 ± 0.9 a

Note: Data are means ± SE (n = 3). Different letters within a column mean significant differences at 5%. 0 d, 3 d and 7 d represent the day of defoliation, three and seven days after defoliation, respectively

**Fig. 3:**
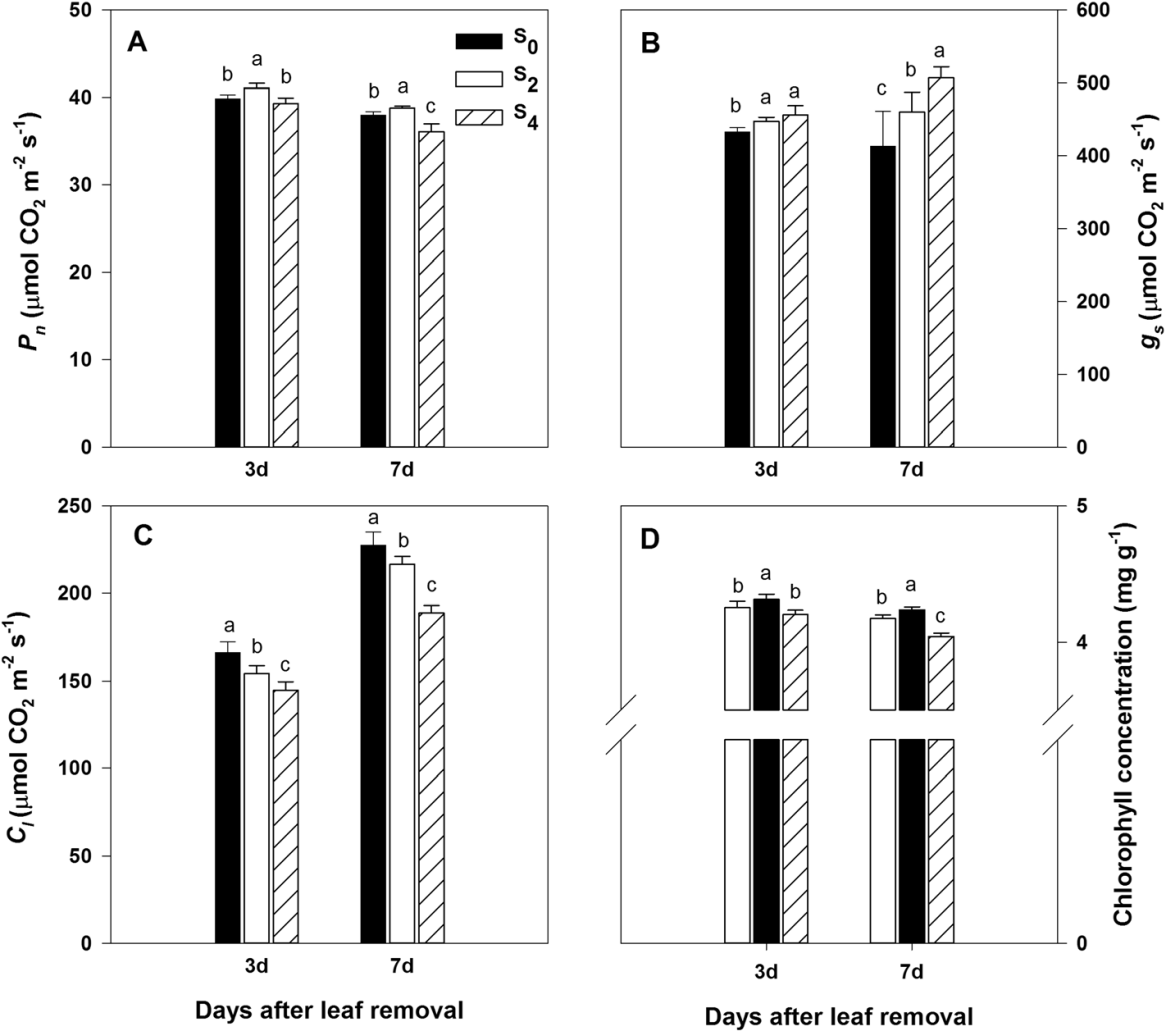
Effects of leaf removal on gas exchange parameters and chlorophyll concentration at three and seven days after defoliation. **a**, **b**, **c** and **d** represent net photosynthetic rate (P_n_), stomatal conductance (g_s_), intercellular CO_2_ concentration (C_i_) and chlorophyll concentration, respectively. S_0_ refers to control (no leaf removal); S_2_ refers to the removal of two leaves and S_4_ refers to the removal of four leaves from top of the plant. Data represent means ± SE (n = 3). Different small letters in each group indicate significant differences at *P* ≤ 0.05

Leaf removal treatments also affected gas exchange parameters of ear leaves (Fig. 3). Net photosynthesis rate (P_n_) and stomatal conductance (g_s_) were significantly increased in S_2_ compared to S_0_, whereas intercellular CO_2_ concentration (C_i_) was significantly decreased after leaf removal. In S_4_ plants, P_n_ was not significantly changed compared to the control at three days after leaf excising, but decreased significantly at seven days after leaf excising. Besides, chlorophyll content had the same trend as P_n_ in response to different leaf removal levels (Fig. 3d).

Table 2 showed the dynamic activities of SOD, POD and content of MDA in maize ear leaves after defoliation. The activities of SOD and POD decreased obviously after leaf removal in both stages compared to the control, and these indices decreased faster (*P* ≤ 0.05) for S_4_ treatment. In contrast, MDA content was increased in both treatments compared to the control.

### Identification of differentially accumulated proteins

The mass error of all the identified peptides met the requirements (centered at 0 and set within 10 ppm). Besides, most peptides were distributed in 8–20 amino acid residues (sample preparation reached the standard). After merging data from three biological replicates, a total of 3586 proteins were identified, and the repeatability of the three replicates were tested using Person’s correlation coefficient (Additional file 2: Figure S1). We considered a ratio of > 1.3 to indicate up-regulation and a ratio of < 0.77 (1/1.3) to indicate down-regulation (*P* ≤ 0.05). Using these two criteria, we identified differentially abundant proteins in leaves subjected to leaf removal. We identified 239 increased proteins and 99 decreased proteins between the S_2_ and S_0_ treatments, and 71 increased proteins and 42 decreased proteins between the S_4_ and S_0_ treatments (Additional file 3: Table S2 and Additional file 4: Table S3).

### Bioinformatic analysis of differentially abundant proteins between the S_2_ and S_0_ treatments

**Fig. 4:**
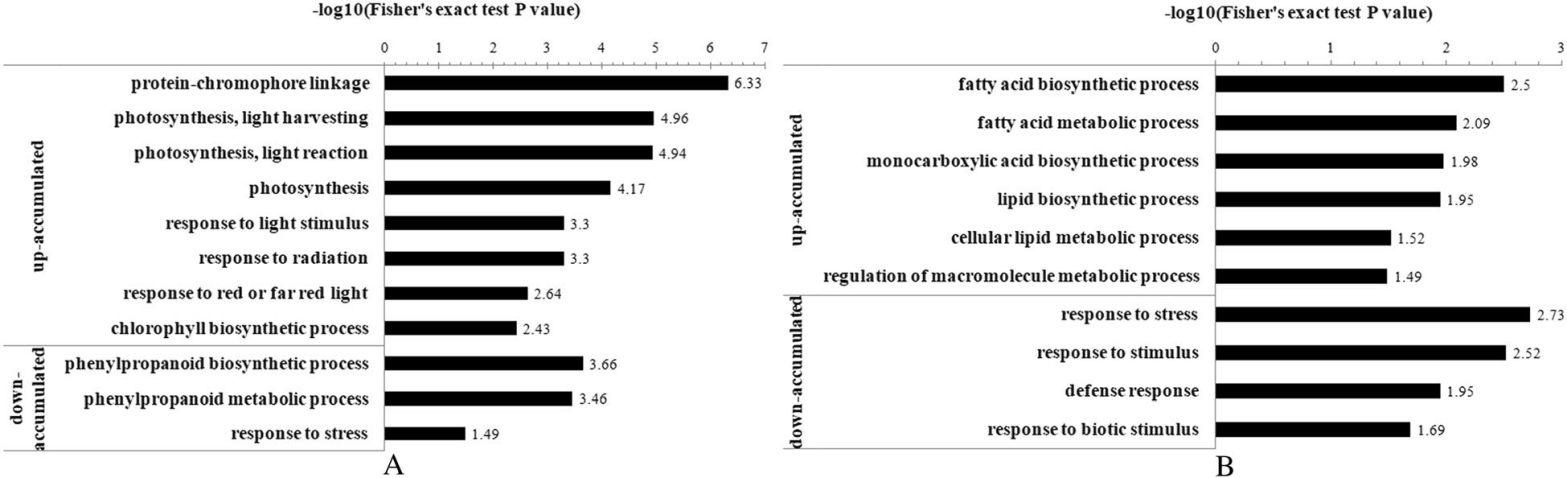
The specific Gene ontology (GO) terms related to physiological changes of differential abundance proteins obtained at three days after defoliation. **a** represents up-accumulated and down-accumulated proteins with S_2_ treatment compared to S_0_ treatment; **b** represents up-accumulated and down-accumulated proteins with S_4_ treatment compared to S_0_ treatment. S_0_ refers to control (no leaf removal); S_2_ and S_4_ refer to the removal of two and four upper leaves, respectively

To identify the significantly enriched GO functional groups of differentially expressed proteins, GO annotation was conducted. The up-accumulated proteins with S_2_ treatment were strongly enriched into 30 functional groups compared with S_0_ (Additional file 5: Figure S2A), of which biological processes, cellular components and molecular functions accounted for 14, 8, and 8 GO terms, respectively. We found that 21 proteins that were up-accumulated in S_2_ compared with S_0_ played roles in photosynthesis (Fig. 4a), including “protein-chromophore linkage”, “photosynthesis, light harvesting”, “photosynthesis, light reaction”, “photosynthesis”, “response to light stimulus”, “response to radiation”, “response to red or far red light”, and “chlorophyll biosynthetic process”. Proteins that were down-accumulated in S_2_ compared with S_0_ were strongly enriched into 23 functional groups (Additional file 5: Figure S2B), of which biological processes, cellular components, and molecular functions accounted for 14, 1, and 8 GO terms, respectively. We found that 13 proteins of the down-accumulated proteins were involved in disease/defence categories (Fig. 4a), including “phenylpropanoid biosynthetic process”, “phenylpropanoid metabolic process”, and “response to stress”.

### Bioinformatic analysis of differentially abundant proteins between S_4_ and S_0_ treatments

Proteins that were up-accumulated in S_4_ compared with S_0_ were strongly enriched into 22 functional groups (Additional file 5: Figure S2C), of which biological processes and molecular functions accounted for 14 and 8 GO terms, respectively. We found that 8 up-accumulated proteins were involved in fatty acid metabolism (Fig. 4b), including “fatty acid biosynthetic process”, “fatty acid metabolic process”, “monocarboxylic acid biosynthetic process”, “lipid biosynthetic process”, “cellular lipid metabolic process,” and “regulation of macromolecule metabolic process”. The proteins that were down-accumulated in S_4_ compared with S_0_ were strongly enriched into 23 functional groups (Additional file 5: Figure S2D), of which biological processes, cellular components and molecular functions accounted for 14, 1 and 8 GO terms. Besides, 10 down-accumulated proteins, which similar to the two leaves removal treatment, were enriched in disease/defence (Fig. 4b), including “response to stress”, “response to stimulus”, “defence response”, and “response to biotic stimulus”.

### qRT-PCR analysis of the expression of genes between treatments and control

We next assayed whether leaf removal treatment had effects on the relative expression of genes encoding proteins changed in S_2_ and S_4_. Quantitative RT-PCR was employed to determine seventeen genes' (*gpm571*, *LOC100273752*, *LOC100284847*, *LOC100281879*, *LOC100282512*, *lhcb6*, *pco103778a*, *psbB*, *psbC*, *Lhcb5–1*, *ACC1*, *LOC100281026*, *LOC100383323*, *LOC103634525*, *gpm853*, *Zlp*, *and LOC100280979*) relative expression in S_2_ and S_4_. In accordance with the protein results, the relative expression of genes involved in the photosynthesis pathways were mostly increased in S_2_ treatment (Additional file 6: Figure S3B), and the expression of genes involved in the fatty acid metabolism were increased in S_4_ treatment (Additional file 6: Figure S3D). Moreover, the relative expressions of defense-related genes were decreased in both S_2_ and S_4_ (Additional file 7: Figure S4B).

## Discussion

### Leaf removal affected the grain yield and physiological parameters of maize

The maize grain yield mainly depends on photosynthesis production by leaves after silking, and the subsequent biomass allocation to kernels [28]. An optimized canopy structure can enhance the light utilization of plants, inhibit protein degradation in leaf and maximize grain yield [29, 30]. In our study, S_2_ enhanced the light transmission rate of both ear and bottom layers (Fig. 1), which enabled the leaves in lower canopy to obtain more light energy and achieve a higher grain yield ultimately [31]. Although LAI decreased (Table 2), Chl content and net photosynthetic rate of ear leaf were enhanced after two leaves removal [12], which may account to the positive compensatory effect of plants [17]. On the contrary, a higher amount of leaf removal (S_4_) resulted in a significant decrease in grain yield compared to the control, which might be due to the insufficient sources to favor the formation of assimilates after four leaves removal. Reactive oxygen species (ROS) are toxic molecules which can cause early senescence and ultimately cell death, and antioxidant enzymes play important roles in detoxifying ROS [32]. In our research, the activities of antioxidant enzymes reduced, whereas the MDA content increased significantly in S_2_ and S_4_ compared to control (Table 2), which demonstrated the extend of peroxidation of membrane lipid was aggravated due to leaf removal after silking. These different physiological reactions between two and four leaves removal in this research indicated that the degree of defoliation affected maize production involved diverse processes. In order to obtain deeper insight into the nature of leaf removal, we focused on a number of proteins involved in notable function categories.

### Two leaves removal enhanced the expression of photosynthesis-related proteins

Chlorophyll molecules are important photoreceptor pigments that absorb light energy and transfer electrons into the photosynthesis reaction centre [33]. Magnesium chelatase catalyses the magnesium-insertion process in the synthesis of Chl. The mutant gene *GUN5* encodes the Mg-chelatase H (Chl H) subunit of Mg-chelatase, which determines the pale phenotypes of this mutant [34]. Magnesium-protoporphyrin IX monomethyl ester (oxidative) cyclase (Chl 27), which is involved in chlorophyll biosynthesis, catalyses the formation of protochlorophyllide. In our study, one Chl H (K7U7W9) and one Chl 27 (K7USR3) were observed to be up-accumulated with S_2_ treatment compared to S_0_ treatment, which may account for the increase of Chl content in ear leaves with S_2_ treatment (Fig. 3d).

Photosynthesis comprises two sets of reactions: photosynthetic electron-transfer reaction and carbon-fixation reaction. In the current research, we found that removing two leaves affected a series of proteins involved in this process (Additional file 3: Table S2). Photosynthetic electron-transfer reaction involves three key events. Firstly, the antenna complexes capture photons and produce high-energy electron. Next, photosystem II (PSII) catalyzes light-driven oxidation of water, releasing oxygen and electrons in this process. Then, the electrons take part in ATP synthesis via the electron transport chain. Finally, the electrons are transferred to photosystem I (PSI) to produce NADPH [35]. We identified a series of proteins involved with this process, including chlorophyll a-b binding proteins (CABs; A0A096RF43, A0A096RM67, A0A096UJK9, A0A096S5Z5, B4FV94, B4FXB0, B6SZT9, B6T892, K7TXI5, and Q41746), PSII reaction centre protein (P05641, P24993, and P48187), oxygen-evolving enhancer protein (A0A096U686), cytochrome oxidase (A0A096U038 and K7UZJ0), cytochrome (A0A096Q1T0 and B6UBZ9), plastocyanin (B6SSB9), PSI assembly protein (A0A096TR75), PSI reaction centre subunit (B4G1K9), ferredoxin (B6TVC7), ATP synthase (K7VI25, K7VN08, P00835, P17344, and P48186) and F1F0-ATPase inhibitor protein (B6T5U0). In plants, CABs can capture light and transfer the excitation energy to PSI and PSII, which plays a central role in the light-harvesting complex (LHC). However, numerous environmental stressors can affect the expression of CABs [36, 37]. In the current study, ten CABs were identified and were all up-accumulated with S_2_ treatment. As the accumulation of CABs, PSI, and PSII can be regulated by the Chl content [38], the up-regulation of CABs in the ear leaves may relate to the increase in Chl content with S_2_ treatment in this study.

The plant PSII core complex has about 20 subunits, consisting of individual proteins and protein complexes [39, 40]. The present study identified four PSII reaction centre proteins as up-accumulated with S_2_ treatment, including one PSII CP47 reaction centre protein (PsbB), one PSII CP43 reaction centre protein (PsbC), one PSII reaction centre protein H (PsbH), and one oxygen-evolving enhancer protein 3–1 (OEE3). PsbB and PsbC, which comprise the PSII reaction centre, play important roles in water splitting [39, 41]. The down-regulation of these two proteins can completely destroy the oxygen-forming capacity of plants [41]. PsbB protein also binds several small transmembrane subunits, except pigments [40, 42]. PsbH is a single transmembrane helix subunit that binds within the PsbB protein as a small subunit, and it plays a key role in the proper functioning of PSII and its stable assembly. In this work, the expression of PsbH was increased with S_2_ treatment compared with the control. Based on previous research [43], we postulated that increased accumulation of PsbH can stabilise the PSII core and promote the PSII electron transfer between the quinone acceptors QA and QB, to some extent. OEE proteins help to increase the efficiency of the oxygen-evolving complex [41] and several proteomics studies have shown that the abundance of OEE protein in plants is affected by stresses, nevertheless the underlying mechanism remains unknown [44, 45]. In our study, this protein was up-accumulated with S_2_ treatment. The function of OEE in the oxygen-evolving complex is considered supplementary, therefore, the up-regulation of this protein may represent a mechanism for the optimisation of oxygen-evolving complex. These results could demonstrate that removing two leaves could affect a series of proteins involved in the system of PSII significantly.

Cytochrome c oxidase and cytochrome bc1 complex are located within the inner mitochondrial membrane, and they function as part of the electron transfer complexes. Meanwhile, cytochrome b is located within the cytochrome b6f and bc1 complexes as part of the electron transport chain [46]. Otherwise, plastocyanin is a copper-containing protein that can receive the electrons from the reduced cytochrome b6f and transmit them to PSI complexes in the photosynthetic electron transfer chain. There are few reports on the change in the abundances of these proteins involved in photosynthesis in response to leaf removal. In our study, the abundances of cytochrome c oxidase and cytochrome bc1 complex were increased but the abundances of cytochrome b and plastocyanin were decreased with S_2_ treatment. These conflicting results may attribute to that cytochrome b is one part of cytochrome bc1, and further studies are required to explore the underlying reasons.

PSI comprises pigment protein super-complexes in higher plants that have approximately 19 subunits [47, 48]. Similar to PSII mentioned above, PSI complexes are also related to a light harvest antenna (LHCI). In our study, the PSI reaction centre proteins were up-accumulated with S_2_ treatment. Moreover, ferredoxin (Fdx), a reducing agent that catalyses the formation of NADPH using NADP^+^ [49] was more abundant in S_2_ treatment than in the control. Previous studies also showed that PSI can use the light energy collected in LHCI to generate reduced ferredoxin using plastocyanin oxidation [47, 48, 50]. This result may account for the decreased abundance of plastocyanin mentioned above.

ATP synthase, which involved in photosynthetic electron-transfer reaction, plays a key role in the non-photochemical quenching of photosynthesis through reactive oxygen species (ROS)-promoted photo inhibition [51]. All differentially expressed ATP synthase was up-accumulated with S_2_ treatment. Moreover, we also found that one ATPase inhibitor protein was down-accumulated with S_2_ treatment. The high levels of ATPase and low levels of ATPase inhibitor protein that we observed in maize ear leaves with the two-leaf removal treatment may result in increased ATP synthesis, thereby decreasing ROS-promoted photo inhibition.

All the proteins discussed above were involved in photosynthetic electron-transfer reaction, and were more abundance in S_2_ treatment compared to S_0_. In concordance with these results, leaf net photosynthetic rate increased apparently in S_2_ treatment in our research (Fig. 3a). Moreover, qRT-PCR results showed that a series of genes which participated in photosynthesis pathway were also more expressed in S_2_ compared to S_0_ (Additional file 6: Figure S3B). The combined results in this study supported the conclusion that two leaves removal enhanced the expression of photosynthesis-related proteins and hence increased the capacity of leaf photosynthesis.

### Four leaves removal increased activities of key enzymes associated with fatty acid metabolism

Unlike with the two-leaf removal treatment, the up-accumulated proteins from the removal of four leaves were strongly enriched into metabolism and secondary metabolism categories. Fatty acids are important in plants because, when metabolised, they produce many ATP molecules. In the fatty acid elongation process, the initial and rate-limiting step is catalysed by the membrane-bound 3-ketoacyl-CoA synthase, which was first identified in *Arabidopsis thaliana* [52]. Acetyl-CoA carboxylases (ACCs) catalyse the formation of malonyl-CoA using acetyl-CoA, and malonyl-CoA is an important substrate in de novo lipogenesis [53]. Acyl carrier protein (ACP) is an independent protein in dissociative type II fatty acid synthase (FAS) found in plants and other organisms [54, 55]. ACP plays a central role in the FAS system by shuttling acyl chain intermediates in its hydrophobic cavity to various enzymes. We identified one acetyl-CoA carboxylase 2, one acyl carrier protein, and one 3-ketoacyl-CoA synthase with S_4_ treatment; their abundances were all increased relative to the control. Besides, qRT-PCR results also showed that two genes (*ACC1*, *LOC100281026*) related to fatty acid metabolism were more expressed in S_4_ treatment compared to S_0_ (Additional file 6: Fig. S3D). These results indicated that removing four leaves can promote the activity of fatty acid metabolism in maize ear leaves.

### Both leaf removal treatments decreased the expression of defense-related proteins

Plants lack of animals’ immune system. Instead, plants have evolved a set of defence mechanisms to protect themselves when attacked by pathogens under natural conditions [56]. For example, phenylpropanoids play central roles in many aspects of the plant responses to abiotic and biotic stimuli and are becoming important indicators in plant’s stress responses to light changing [57]. As previous research shows, decreasing the phenylpropanoid biosynthesis rate can significantly lower plant resistance [58, 59]. In our study, we identified three proteins (B4FQP4, K7VC35, and Q6VWJ0) related to the phenylpropanoid biosynthetic process, which abundances were decreased with S_2_ treatment. Besides, we also found that a series of proteins (A0A096RTN1, A0A096T686, A0A0B4J3G7, B4FA32, B4FR89, B6SIF0, B6SQM0, C0HGH7, K7VC35, K7VH58, and P33679) involved with the defence categories were down-accumulated with S_2_ treatment. Similar results have been reported in rice that received a stress [60].

Moreover, a series of proteins that play roles in plant defences were identified in S_4_ treatment. Many host proteins are induced in plants during pathogen attacks, and the majority are pathogen-related (PR) proteins. PR proteins are categorised into 17 families (PR1 to PR 17) by their structures and biological activities [61]. Among these families, PR10 proteins play vital roles in resisting biotic and abiotic stresses [62]. In this research, we identified one PR protein 10b down-accumulated with S_4_ treatment compared to the control, which suggested that leaf removal may also affect the capability of maize to resist stresses.

The level of ROS in plants always increases rapidly in response to abiotic or biotic stresses [63, 64]. Peroxidases catalyse the reduction of peroxide or hydroperoxides using an oxidised donor substrate (typically a thiol), thereby regulating H_2_O_2_ levels. In our study, we identified two peroxidases (A0A0B4J3G7 and B4FA32) that were differentially expressed with S_4_ treatment. The down-regulation of these two peroxidases indicates that changes in ROS levels occur in ear leaves with S_4_ treatment. As investigations in plants under different abiotic stresses [63–66], we supposed that peroxidase was damaged after four leaves removing and therefore cannot detoxify ROS-induced lipid peroxidation products.

Asr proteins function in response to abiotic stresses in plants [67–71]. Therefore, the down-regulation of Asr protein (A8IK79) in ear leaves with S_4_ treatment indicated that removing four leaves weakened the tolerance of maize to abiotic stress. Zeamatin was first identified in corn seeds with high amino acid homology to PR-5 proteins. Besides, it has potent antifungal activity against a number of plant pathogens [72]. We identified one zeamatin, the expression of which was decreased significantly in ear leaves with S_4_ treatment. Otherwise, the activities of antioxidant enzymes were found significantly decreased in both treatments (Table 2). In addition, some genes encoded the defence-related proteins were also less expressed in both S_2_ and S_4_ treatments compared to S_0_ (Additional file 7: Figure S4B). Therefore, to a certain extent, both two and four leaves removal may affect the defensive system of maize in both physiological and molecular level.

Plant yield production and defense response are trade off regarding the energy distribution. Due to the contrasting yield production between treatment S_2_ and S_4_, it would be really interesting to test the effects of different defoliation numbers (one, three or more leaves removal) upper the ear leaves. That would be conducive to fully elucidate the effects of leaf removal in maize.

## Conclusions

Based on our study, we demonstrated that different defoliation levels had contrastive effects on maize. The canopy light transmission rate was strengthened and proteins related to photosynthetic electron-transfer reaction were up-regulated significantly for treatment S_2_, which improved the leaf photosynthetic capacity, and obtained a higher grain yield consequently. In contrast, S_4_ decreased the grain yield and increased the expressions of proteins and genes associated with fatty acid metabolism. Besides, both S_2_ and S_4_ treatments exaggerated the defensive response of maize in physiological and proteomic level. Although further studies of leaf removal are required, the results in our study provide new insights to the effects of leaf removal in maize.

## Additional files

Additional file 1: **Table S1.** Primers used in quantitative RT-PCR in this study. (DOCX 31 kb)

Additional file 2: **Figure S1.** The Pearson correlation analysis of the three replicates of each treatment. S_0_ refers to control (no leaf removal); S_2_ and S_4_ refer to the removal of two and four uppermost leaves, respectively. (TIF 1038 kb)

Additional file 3: **Table S2.** Differences in protein abundances between the two-leaf removal treatment (S_2_) and the control (S_0_). (DOCX 85 kb)

Additional file 4: **Table S3.** Differences in protein abundances between the four-leaf removal treatment (S_4_) and the control (S_0_). (DOCX 48 kb)

Additional file 5: **Figure S2.** Gene ontology (GO) classification of differentially accumulated proteins. (**A**) Up-regulated proteins with S_2_ treatment compared to S_0_ treatment; (**B**) down-regulated proteins with S_2_ treatment compared to S_0_ treatment; (**C**) up-regulated proteins with S_4_ treatment compared to S_0_ treatment; and (**D**) down-regulated proteins with S_4_ treatment compared to S_0_ treatment. S_0_ refers to control (no leaf removal); S_2_ and S_4_ refer to the removal of two and four uppermost leaves, respectively. (PDF 199 kb)

Additional file 6: **Figure S3.** Effects of leaf removal on relative expression of photosynthesis related proteins (**A**), the corresponding encoding genes (**B**) in S_2_ and relative expression of fatty acid metabolism related proteins (**C**) and the encoding genes (**D**) in S_4_, compared to S_0_ respectively. The gene candidates are selected by proteins which accumulate in photosynthesis and fatty acid biosynthetic process terms in Fig. 4. S_0_ refers to no leaf removal (control); S_2_ and S_4_ refer to the removal of two or four uppermost leaves, respectively. Data are means ± SE (*n* = 3). ^*^ indicates the significant difference at *P* ≤ 0.05 level. (PDF 57 kb)

Additional file 7: **Figure S4.** Effects of leaf removal on relative expression of defense related proteins (**A**) and the encoding genes (**B**) in S_2_ and S_4_ compared to S_0_. S_0_ refers to no leaf removal (CK); S_2_ and S_4_ refer to the removal of two or four leaves, respectively. Data are means ± SE (n = 3). Different lowercase letters indicate the significant difference at *P* ≤ 0.05 level. (PDF 16 kb)

## Abbreviations

ACCs: acetyl-CoA carboxylases
ACP: acyl carrier protein
CAB: chlorophyll a-b binding protein
Chl: chlorophyll
C_i_: intercellular CO_2_ concentration
DAS: days after silking
FAS: fatty acid synthetase
Fdx: ferredoxin
GO: Gene Ontology
g_s_: stomatal conductance
LHCI: light harvest antenna
MDA: malondialdehyde
OEE: oxygen-evolving enhancer protein
P_n_: net photosynthetic rate
POD: peroxidase
PR: pathogen-related
ROS: reactive oxygen species
SOD: superoxide dismutase
TMT: tandem mass tags
