# Supplementary material for: Physiological and proteome studies of maize (Zea mays L.) in response to leaf removal under high plant density
Source: BMC Plant Biol. 2018 Dec 29;18:378. doi: 10.1186/s12870-018-1607-8 (PMC6310946; doi:10.1186/s12870-018-1607-8)
Supplement: Supplementary file 1 — Table S1. Primers used in quantitative RT-PCR in this study. (DOCX 31 kb) [file 12870_2018_1607_MOESM1_ESM.docx]

**Table S1.** Primers used in quantitative RT- PCR in this study.

| **Protein accession** | **Gene ID** | **Primers** |
| --- | --- | --- |
|  |  |  |
| **Photosynthesis-related proteins** |  |  |
| B4FV94 | *gpm571* | F: GCCTTCCTTGGCCACTCTA |
|  |  | R: CGAACCACCGCAGGTTCT |
| B4FXB0 | *LOC100273752* | F: CGTGGATCCCCGCCTTCAAGA |
|  |  | R: CACTTGAGGAACGCCGGGT |
| B4G1K9 | *LOC100284847* | F: GCCCGTCGCTGGTGATCA |
|  |  | R: GCACCTGCTTCGCCACGT |
| B6SZT9 | *LOC100281879* | F: GCGCCGCACCGTCAAGA |
|  |  | R: GGGAACTCGCCGGTCAGGTA |
| B6T892 | *LOC100282512* | F: CCGACAGGCCGATCTGGT |
|  |  | R: GCAGGCTCTCCGGGTCA |
| K7TXI5 | *lhcb6* | F: GTCGTGGATCCCGGCCATCAA |
|  |  | R: CCTCCCGGTACCACTTGAGGA |
| K7VXL2 | *pco103778a* | F: GGCTGGTATGGAAGGGACTGTGA |
|  |  | R: GACAGGATCATGGCCAGGTGTCA |
| P05641 | *psbB* | F: GGCAGCTATCTGGCATTGGGTATA |
|  |  | R: GCACCAAAGCCAAAACAAGCCA |
| P48187 | *psbC* | F: GGGCTGGGAATGCCAGACTTA |
|  |  | R: GCGGAAGTAAAATCAACCCTTGTTCA |
| Q41746 | *Lhcb5-1* | F: GATCTACCTGCCGGATGGGCT |
|  |  | R: GTCCTCTGGCTTCTTGCCCA |
| **Fatty acid metabolism-related proteins** |  |  |
| A0A096UDB7 | *ACC1* | F: CCCTGGCTCCCCAATATTTGTCA |
|  |  | R: GCACACTGCAATCACGACTGTGA |
| B4FV78 | *LOC100281026* | F: CCATCTCTGCCAACGTCGGCTA |
|  |  | R: GAGACCACAATCCGGCCTGGTA |
| **Defense-related proteins**  A0A0B4J3G7 | *LOC100383323* |  |
|  |  | F: GCTCAGGCTGCACTTCCATGA |
| A0A096RTN1 | *LOC103634525* | R: GATGTTGGCGATGACGTCGA |
|  |  | F: GCGTCAGGCAGTTCAACTTCA |
|  |  | R: GACTCCACCTTGACGTGCGA |
| B6SQM0 | *LOC100280979* | F: GCCAAGGTGGAGCTCGTCA |
|  |  | R: GCCATCAATGGCTTCTGCCT |
| P33679 | *Zlp* | F: GTACGCGCTGAAGCAGTTCA |
|  |  | R: GCACGCGTTGTTGCACA |
| B4FA32 | *gpm853* | F: GTGCGGACCACCATGCTCA |
|  |  | R: CGACGTCGTCCAGAAGAATGGA |
|  | ***Zmactin*** | F: CGATTGAGCATGGCATTGTCA |
|  |  | R: CCCACTAGCGTACAACGAA |
|  |  |  |
|  |  |  |
|  |  |  |
|  |  |  |

Notes: F- forward primer; R- reverse primer.
