# Supplementary figures and images for: Physiological and proteome studies of maize (Zea mays L.) in response to leaf removal under high plant density
Source: BMC Plant Biol. 2018 Dec 29;18:378. doi: 10.1186/s12870-018-1607-8 (PMC6310946; doi:10.1186/s12870-018-1607-8)

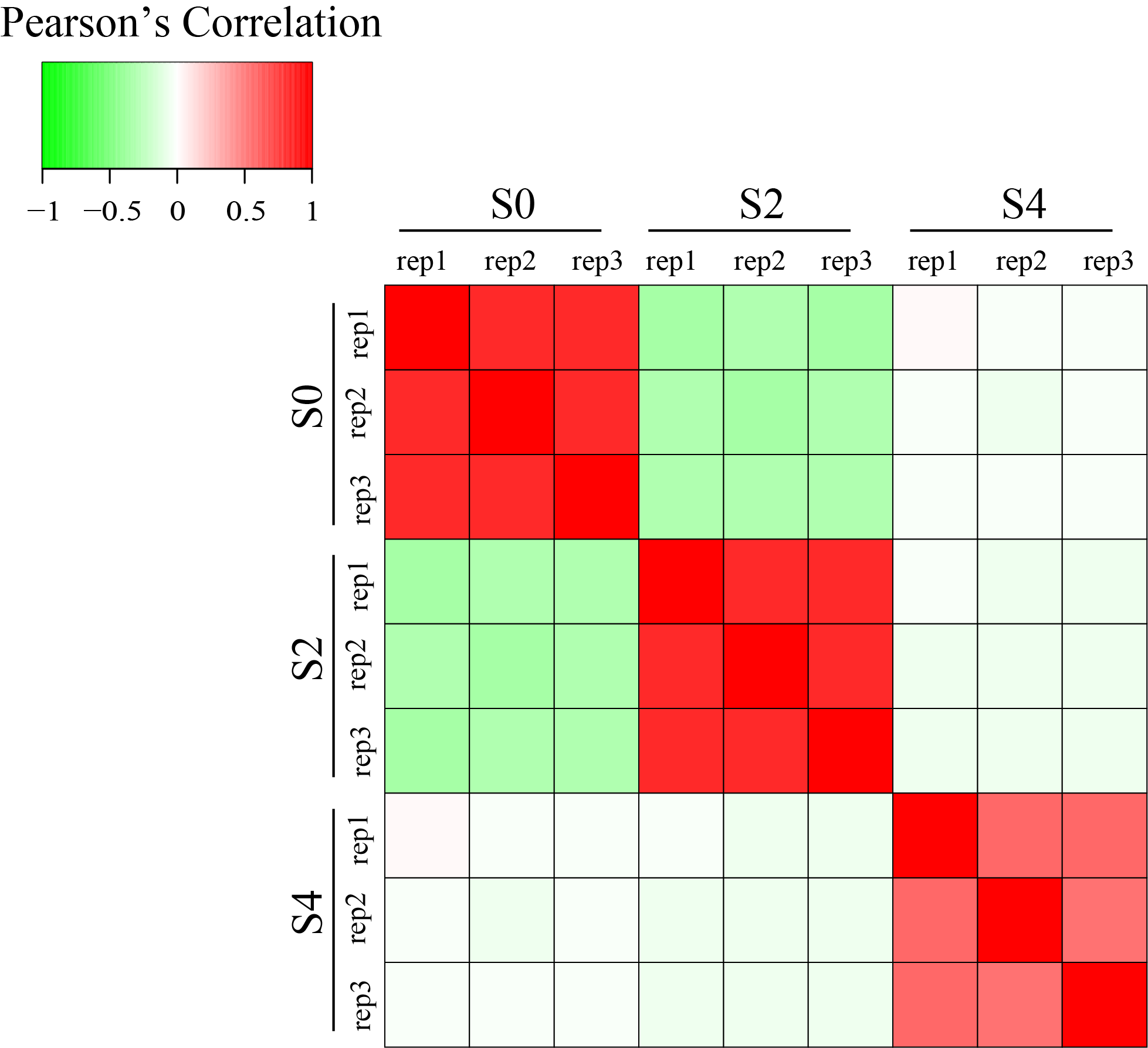

Supplement: Supplementary file 2 — Figure S1. The Pearson correlation analysis of the three replicates of each treatment. S0 refers to control (no leaf removal); S2 and S4 refer to the removal of two and four uppermost leaves, respectively. (TIF 1038 kb) [file 12870_2018_1607_MOESM2_ESM.tif]
