# Supplementary material for: Physiological and proteome studies of maize (Zea mays L.) in response to leaf removal under high plant density
Source: BMC Plant Biol. 2018 Dec 29;18:378. doi: 10.1186/s12870-018-1607-8 (PMC6310946; doi:10.1186/s12870-018-1607-8)
Supplement: Supplementary file 3 — Table S2. Differences in protein abundances between the two-leaf removal treatment (S2) and the control (S0). (DOCX 85 kb) [file 12870_2018_1607_MOESM3_ESM.docx]

**Table S2** Differences in protein abundances between the two-leaf removal treatment (S_2_) and the control (S_0_).

| **Protein** | **Protein description^b^** | **Ratio** | **P** | **pI** | **MW^c^** | **Score** | **AASC^d^** | **Number of** |
| --- | --- | --- | --- | --- | --- | --- | --- | --- |
| **accession^a^** |  | **S_2_/S_0_** | **value** |  | **[kDa ]** |  | **[%]** | **peptide** |
| **Up-regulation** |  |  |  |  |  |  |  |  |
| A0A096RX02 | Calmodulin1 | 2.8 | 0.0004 | 4.57 | 25519 | 278 | 16.7 | 6 |
| B6U3H3 | CP12-1 | 2.59 | 0.0282 | 4.73 | 16777 | 128 | 40.7 | 5 |
| A0A096SXA0 | Putative plastid ribosomal protein L19 | 2.56 | 0.0054 | 10.12 | 26630 | 48 | 6.8 | 3 |
| B4FV78 | Acyl carrier protein | 2.43 | 0.0112 | 5.24 | 17616 | 150 | 16.3 | 3 |
| A0A096SF87 | Not assigned | 2.4 | 0.0000 | 5.23 | 20863 | 144 | 26.4 | 6 |
| A0A096QT00 | Putative potassium/proton antiporter-like protein | 2.32 | 0.0001 | 5.14 | 100956 | 45 | 3 | 2 |
| B4FDK7 | Not assigned | 2.32 | 0.0018 | 5.54 | 46616 | 70 | 8.4 | 2 |
| A0A096U038 | Cytochrome c oxidase subunit Vb | 2.27 | 0.0002 | 5.11 | 18196 | 48 | 8.3 | 2 |
| K7V8I9 | Not assigned | 2.24 | 0.0222 | 5.76 | 42543 | 47 | 6.3 | 2 |
| K7TL05 | 14-3-3-like protein GF14-12 | 2.22 | 0.0002 | 4.75 | 34079 | 881 | 65.5 | 43 |
| B6U284 | 14-3-3-like protein | 2.15 | 0.0005 | 4.78 | 33773 | 441 | 57.4 | 26 |
| A0A096PUI0 | "Probable signal recognition particle 43 kDa protein, chloroplastic" | 2.14 | 0.0124 | 4.79 | 50278 | 44 | 2.6 | 1 |
| B4FR29 | "Rubisco accumulation factor 1, chloroplastic" | 2.1 | 0.0002 | 5.29 | 53522 | 93 | 8.9 | 5 |
| B4FA21 | Purple acid phosphatase | 2.05 | 0.0162 | 5.53 | 75590 | 74 | 6.2 | 3 |
| K7TQQ1 | OSJNBb0070J16.4 protein | 2.03 | 0.0000 | 6.26 | 28141 | 135 | 17.4 | 6 |
| K7TNK3 | Not assigned | 2.01 | 0.0001 | 4.68 | 180172 | 52 | 4.8 | 6 |
| K7V7C2 | Not assigned | 2 | 0.0011 | 4.79 | 27381 | 46 | 12.2 | 3 |
| A0A096RB78 | Putative heme binding protein cemA | 1.99 | 0.0000 | 5.03 | 54610 | 79 | 6.4 | 1 |
| B6TH64 | 40S ribosomal protein S9 | 1.94 | 0.0000 | 10.29 | 26227 | 87 | 28.5 | 10 |
| A0A096Q176 | Putative steroid 22-alpha-hydroxylase | 1.93 | 0.0018 | 8.8 | 75901 | 208 | 8.9 | 5 |
| A0A096QPQ8 | Not assigned | 1.91 | 0.0000 | 6.4 | 24156 | 23 | 6.1 | 1 |
| C3UZ63 | HSP protein | 1.9 | 0.0000 | 4.98 | 98930 | 195 | 18 | 13 |
| K7VMA9 | 30S ribosomal protein S1 | 1.9 | 0.0009 | 4.68 | 47689 | 164 | 25.9 | 12 |
| B6TFS9 | 14-3-3-like protein A | 1.89 | 0.0026 | 4.86 | 31697 | 242 | 33.2 | 19 |
| C4J6C7 | Eukaryotic translation initiation factor 1A | 1.89 | 0.0000 | 5.08 | 20173 | 51 | 14.6 | 2 |
| K7UK77 | OSJNBb0070J16.4 protein | 1.89 | 0.0440 | 5.43 | 45516 | 102 | 11.8 | 7 |
| A0A096R1R9 | "Nucleolar protein NOP5, putative, expressed" | 1.88 | 0.0000 | 7.23 | 62752 | 100 | 6.4 | 5 |
| K7TGX4 | Not assigned | 1.88 | 0.0010 | 6.75 | 19515 | 97 | 21 | 3 |
| B6T671 | Not assigned | 1.87 | 0.0001 | 7.75 | 32972 | 225 | 30.9 | 6 |
| C0P9K3 | Inner membrane protein ALBINO3 | 1.87 | 0.0000 | 8.92 | 36965 | 81 | 8.4 | 6 |
| B4FRE3 | Ankyrin repeat domain-containing protein 2 | 1.86 | 0.0026 | 4.57 | 43244 | 139 | 17.5 | 8 |
| A0A096QT89 | "Putative oxidoreductase, aldo/keto reductase family protein" | 1.84 | 0.0000 | 7.15 | 45723 | 67 | 7.6 | 3 |
| A0A096U8E3 | Putative TGB12K interacting protein 3 | 1.84 | 0.0000 | 4.64 | 35820 | 115 | 15.5 | 5 |
| A0A096UHG3 | Not assigned | 1.82 | 0.0006 | 5.48 | 80373 | 479 | 32.3 | 21 |
| A0A096TZN8 | "30S ribosomal protein S16, chloroplastic" | 1.8 | 0.0000 | 9.99 | 15826 | 34 | 13.2 | 2 |
| B4FSN2 | Inner membrane protein ALBINO3 | 1.79 | 0.0058 | 9.3 | 54780 | 118 | 7 | 6 |
| A0A096S1S1 | Expressed protein | 1.78 | 0.0000 | 5.56 | 71316 | 41 | 4.8 | 2 |
| B6TJC7 | Membrane steroid-binding protein 1 | 1.78 | 0.0001 | 5.45 | 30672 | 112 | 6.1 | 2 |
| Q06XS3 | Lipoxygenase | 1.76 | 0.0012 | 6.11 | 114364 | 1697 | 39.6 | 96 |
| K7V3T7 | Stem glycoprotein | 1.75 | 0.0134 | 4.99 | 25869 | 74 | 12 | 3 |
| P24993 | Photosystem II reaction center protein H | 1.75 | 0.0015 | 8.09 | 8928 | 212 | 16.4 | 13 |
| A0A096UDG8 | "50S ribosomal protein L32, chloroplastic" | 1.74 | 0.0000 | 10.41 | 8915 | 29 | 15.5 | 2 |
| K7TQN7 | OSJNBb0065L13.3 protein | 1.74 | 0.0001 | 5.01 | 83669 | 125 | 6.6 | 3 |
| K7V364 | "Hsp90 protein, expressed" | 1.74 | 0.0001 | 5.04 | 104703 | 316 | 9.7 | 8 |
| B6TZ58 | VAMP protein SEC22 | 1.73 | 0.0000 | 9.23 | 24611 | 43 | 4.2 | 2 |
| A0A096RLN4 | Ubiquinol oxidase | 1.71 | 0.0000 | 5.52 | 17159 | 42 | 7.9 | 2 |
| B6T892 | Chlorophyll a-b binding protein | 1.71 | 0.0022 | 5.65 | 31544 | 165 | 27.4 | 6 |
| A0A096SD19 | Putative MAR binding protein | 1.7 | 0.0000 | 4.9 | 82072 | 400 | 27 | 17 |
| A0A096QY80 | 60S acidic ribosomal protein P0 | 1.69 | 0.0052 | 9.5 | 26642 | 148 | 21.8 | 6 |
| B6T927 | Not assigned | 1.67 | 0.0001 | 5.02 | 28430 | 268 | 41.3 | 18 |
| K7U5A5 | 14-3-3-like protein | 1.67 | 0.0000 | 4.8 | 33076 | 744 | 67.9 | 37 |
| B6STX8 | ATP-dependent protease Clp ATPase subunit | 1.66 | 0.0000 | 7.74 | 41326 | 30 | 2.2 | 1 |
| B6SUJ3 | Plastid-specific 30S ribosomal protein 2 | 1.65 | 0.0035 | 6.48 | 34044 | 95 | 14 | 3 |
| A0A096R5D9 | Expressed protein | 1.64 | 0.0006 | 4.59 | 110946 | 348 | 15.2 | 17 |
| A0A096T026 | Not assigned | 1.64 | 0.0000 | 9.43 | 37557 | 33 | 2.6 | 1 |
| B6T7B2 | 40S ribosomal protein S9 | 1.64 | 0.0001 | 10.32 | 26371 | 187 | 35.6 | 12 |
| A0A096T4D3 | 60S ribosomal protein L27 | 1.63 | 0.0009 | 10.58 | 24499 | 117 | 16.8 | 4 |
| A0A096THK1 | 60S acidic ribosomal protein P3 | 1.62 | 0.0000 | 4.45 | 13417 | 53 | 15 | 1 |
| K7TYJ1 | Ribonucleoprotein | 1.62 | 0.0150 | 4.69 | 33106 | 116 | 17.5 | 4 |
| A0A096RTH6 | Heat shock protein | 1.6 | 0.0000 | 5.01 | 98969 | 240 | 15.9 | 12 |
| K7UZJ0 | Cytochrome c oxidase subunit Vb | 1.6 | 0.0001 | 4.94 | 20013 | 74 | 29.3 | 5 |
| Q5EUD5 | Protein disulfide isomerase | 1.6 | 0.0000 | 5.45 | 56203 | 35 | 4.6 | 2 |
| A0A096UHC7 | Putative karyopherin-beta 3 variant | 1.59 | 0.0000 | 4.83 | 87759 | 46 | 2.4 | 2 |
| A0A096RH90 | Not assigned | 1.58 | 0.0207 | 5.69 | 45429 | 50 | 3.4 | 1 |
| A0A096SYW2 | Not assigned | 1.58 | 0.0000 | 5.15 | 40575 | 35 | 4.1 | 1 |
| A0A0B4J3K6 | CHP-rich zinc finger protein-like | 1.58 | 0.0000 | 4.81 | 30451 | 44 | 3.3 | 1 |
| B4FUD0 | Not assigned | 1.57 | 0.0000 | 9.13 | 28382 | 29 | 9.3 | 2 |
| K7TQX2 | Not assigned | 1.56 | 0.0002 | 4.68 | 66267 | 232 | 20.4 | 10 |
| K7UV99 | Putative ABC transporter | 1.55 | 0.0000 | 5.09 | 68670 | 143 | 9.3 | 7 |
| A0A096PWV0 | Non-specific lipid-transfer protein | 1.54 | 0.0004 | 9.27 | 12879 | 302 | 54.6 | 10 |
| K7V0H9 | Riboflavin biosynthesis protein ribAB | 1.54 | 0.0000 | 5.43 | 63483 | 45 | 4.1 | 1 |
| A0A096PWG2 | Serine/threonine-protein kinase SNT7 | 1.53 | 0.0000 | 9.41 | 70027 | 341 | 19.9 | 13 |
| A0A096SXW7 | Expressed protein | 1.53 | 0.0003 | 9.03 | 110920 | 103 | 6.1 | 6 |
| A0A096THE5 | "Probable polyribonucleotide nucleotidyltransferase 1, chloroplastic" | 1.53 | 0.0000 | 5.69 | 108687 | 372 | 15.1 | 13 |
| B4FRG1 | 14-3-3-like protein | 1.53 | 0.0000 | 4.8 | 32848 | 422 | 65 | 32 |
| A0A096RS73 | Expressed protein | 1.52 | 0.0284 | 10.08 | 15839 | 56 | 11.2 | 2 |
| A0A096SAH7 | OSJNBb0065J09.14 protein | 1.52 | 0.0000 | 5.94 | 47011 | 908 | 34.9 | 43 |
| B4FYD8 | Peroxidase | 1.52 | 0.0116 | 5.84 | 37529 | 113 | 10.1 | 2 |
| C0P639 | 60S ribosomal protein L18a | 1.52 | 0.0000 | 10.61 | 26115 | 93 | 19.1 | 3 |
| C4J9W7 | Ubiquinol oxidase | 1.52 | 0.0000 | 5.63 | 42579 | 39 | 5.6 | 2 |
| A0A096PIP9 | "DEAD-box ATP-dependent RNA helicase 3, chloroplastic" | 1.51 | 0.0049 | 6.51 | 87625 | 200 | 16 | 5 |
| B4FKI9 | Not assigned | 1.51 | 0.0000 | 9.26 | 23728 | 43 | 7.8 | 1 |
| B6THU5 | Elongation factor 1-delta 1 | 1.51 | 0.0027 | 4.39 | 29236 | 212 | 36.2 | 11 |
| A0A096PZD2 | "50S ribosomal protein L22, chloroplastic" | 1.5 | 0.0000 | 10.75 | 20741 | 29 | 4.4 | 2 |
| A0A0B4J3F5 | "'putative lipase class 3 family protein, PF01764'" | 1.5 | 0.0002 | 6.29 | 59740 | 870 | 37.6 | 36 |
| B4F925 | Superoxide dismutase | 1.5 | 0.0028 | 7.11 | 29925 | 463 | 22.1 | 16 |
| K7UBT7 | "HEAT repeat, putative" | 1.5 | 0.0000 | 8.29 | 176673 | 49 | 1.7 | 1 |
| A0A096QVW8 | "Divinyl chlorophyllide a 8-vinyl-reductase, chloroplastic" | 1.49 | 0.0045 | 7.68 | 43689 | 144 | 21 | 8 |
| A0A096RCS5 | "Phytoene dehydrogenase, chloroplastic/chromoplastic" | 1.49 | 0.0110 | 5.32 | 64566 | 46 | 2.3 | 1 |
| B4FNZ2 | Tubulin-specific chaperone A | 1.49 | 0.0000 | 5.08 | 16053 | 60 | 48.2 | 3 |
| B6SK48 | Late embryogeneis abundant protein | 1.49 | 0.0090 | 5.96 | 18250 | 85 | 10.6 | 2 |
| B6TBW4 | Proliferation-associated protein 2G4 | 1.49 | 0.0010 | 6.58 | 54145 | 157 | 16.5 | 11 |
| C0PAS9 | "(Uaz270(357)), mRNA" | 1.49 | 0.0000 | 5.19 | 18384 | 35 | 8.2 | 1 |
| K7VI25 | "ATPase 3, putative, expressed" | 1.49 | 0.0006 | 6.23 | 66659 | 123 | 10.4 | 4 |
| B4FNL7 | MAP3K-like protein kinase | 1.48 | 0.0000 | 5.96 | 47926 | 39 | 2.2 | 1 |
| C4J4B7 | Calcium sensing receptor | 1.48 | 0.0000 | 4.88 | 73370 | 518 | 27 | 23 |
| K7UYT6 | Not assigned | 1.48 | 0.0000 | 8.44 | 63060 | 32 | 6.8 | 2 |
| Q41834 | Nucleic acid-binding protein | 1.48 | 0.0024 | 4.6 | 34987 | 189 | 21.8 | 5 |
| A0A096QA11 | Elongation factor 1-delta 1 | 1.47 | 0.0017 | 4.47 | 32214 | 241 | 26.7 | 12 |
| K7TXI5 | Chlorophyll a/b-binding apoprotein CP24 | 1.47 | 0.0018 | 7.9 | 30007 | 991 | 48.4 | 36 |
| P00835 | "ATP synthase epsilon chain, chloroplastic" | 1.47 | 0.0398 | 5.03 | 16869 | 366 | 20.4 | 20 |
| A0A096SHP7 | Expressed protein | 1.46 | 0.0040 | 8.69 | 33273 | 111 | 21.3 | 4 |
| A0A096TL89 | Not assigned | 1.46 | 0.0000 | 13.01 | 31845 | 46 | 4.5 | 2 |
| B4FDE1 | Eukaryotic translation initiation factor 5A | 1.46 | 0.0000 | 5.19 | 21326 | 39 | 18.8 | 5 |
| B4FR80 | Not assigned | 1.46 | 0.0062 | 4.85 | 30914 | 249 | 38.6 | 9 |
| B4FWJ8 | Luminal-binding protein 3 | 1.46 | 0.0012 | 5.13 | 87919 | 382 | 15.4 | 13 |
| B6U237 | Heat shock 70 kDa protein 4 | 1.46 | 0.0023 | 5.28 | 109663 | 114 | 8.3 | 8 |
| K7USQ8 | Sialin | 1.46 | 0.0009 | 8.8 | 59107 | 140 | 10.3 | 3 |
| K7VCA6 | MRP-like ABC transporter | 1.46 | 0.0000 | 7.15 | 178989 | 32 | 1.1 | 2 |
| A0A096TIC5 | Not assigned | 1.45 | 0.0039 | 9.33 | 46945 | 233 | 24.9 | 6 |
| B4FIE5 | Nascent polypeptide-associated complex subunit beta | 1.45 | 0.0030 | 6.62 | 21882 | 45 | 23.6 | 3 |
| K7ULX0 | Long chain acyl-CoA synthetase | 1.45 | 0.0000 | 6.67 | 38272 | 61 | 8.9 | 3 |
| B4FQN6 | Sorbitol transporter | 1.44 | 0.0000 | 7.71 | 59606 | 29 | 3.4 | 2 |
| B8A324 | Retinoid-inducible serine carboxypeptidase | 1.44 | 0.0000 | 5.36 | 56182 | 119 | 9.6 | 3 |
| C0HFM4 | Putative 60S ribosomal protein | 1.44 | 0.0000 | 10.22 | 29800 | 114 | 45.6 | 10 |
| A0A096Q0G2 | Putative AMP-binding protein | 1.43 | 0.0005 | 5.68 | 75950 | 74 | 4.3 | 2 |
| A0A096RF43 | Chlorophyll a/b-binding protein | 1.43 | 0.0262 | 5.56 | 32197 | 434 | 31.9 | 25 |
| B4G1K9 | Photosystem I reaction center subunit V | 1.43 | 0.0346 | 10.09 | 16412 | 127 | 16.9 | 11 |
| C4J917 | "ATP-dependent zinc metalloprotease FTSH 7, chloroplastic" | 1.43 | 0.0031 | 8.51 | 95885 | 99 | 8.7 | 4 |
| K7TNQ6 | Starch synthase IIIb-1 | 1.43 | 0.0004 | 5.41 | 147466 | 46 | 1.8 | 2 |
| P49085 | "Phytoene synthase, chloroplastic" | 1.43 | 0.0000 | 8.81 | 51205 | 51 | 4.4 | 1 |
| A0A096PL90 | Not assigned | 1.42 | 0.0000 | 9.88 | 20037 | 30 | 6.3 | 1 |
| A0A096SAT9 | OSJNBb0034I13.6 protein | 1.42 | 0.0113 | 5.03 | 79402 | 44 | 5.3 | 3 |
| A0A096SCN7 | DnaJ protein family-like | 1.42 | 0.0091 | 5.37 | 50149 | 73 | 3.1 | 2 |
| B6SMV8 | Not assigned | 1.42 | 0.0019 | 8.82 | 11472 | 55 | 18.3 | 2 |
| P17344 | "ATP synthase subunit a, chloroplastic" | 1.42 | 0.0044 | 5.29 | 29625 | 41 | 2.8 | 2 |
| A0A096PQR7 | Putative cytochrome P450 superfamily protein | 1.41 | 0.0217 | 7.66 | 60810 | 154 | 19.1 | 12 |
| A0A096PRE6 | "Probable plastid-lipid-associated protein 3, chloroplastic" | 1.41 | 0.0003 | 4.58 | 43622 | 118 | 15.2 | 9 |
| A0A096SIT0 | Peroxidase | 1.41 | 0.0005 | 5.03 | 36942 | 64 | 9 | 3 |
| A0A096TKH3 | Putative UOS1 | 1.41 | 0.0016 | 9.31 | 77981 | 382 | 19 | 9 |
| B4FV94 | Chlorophyll a-b binding protein 4 | 1.41 | 0.0000 | 6.6 | 29519 | 221 | 19.4 | 9 |
| B6ST57 | Deoxyribodipyrimidine photolyase family protein | 1.41 | 0.0079 | 7.99 | 88700 | 136 | 11.3 | 9 |
| K7VEN2 | Not assigned | 1.41 | 0.0001 | 9.27 | 50705 | 149 | 17.7 | 7 |
| A0A096QLJ5 | "Probable zinc metalloprotease EGY3, chloroplastic" | 1.4 | 0.0000 | 5.38 | 70803 | 24 | 1.5 | 1 |
| A0A096TT23 | Cystatin2 | 1.4 | 0.0029 | 9.6 | 14800 | 94 | 14.2 | 2 |
| B4FUC4 | Salt stress root protein RS1 | 1.4 | 0.0000 | 5.02 | 27700 | 43 | 14.9 | 3 |
| B4FXB0 | Chlorophyll a-b binding protein CP24 | 1.4 | 0.0079 | 7.9 | 30531 | 876 | 46.2 | 33 |
| B6TXF9 | OSJNBb0065J09.14 protein | 1.4 | 0.0042 | 9.62 | 65128 | 42 | 4.6 | 2 |
| C0HFG8 | 3-ketoacyl-CoA synthase | 1.4 | 0.0000 | 9.19 | 65926 | 36 | 2.9 | 2 |
| P48187 | Photosystem II CP43 reaction center protein | 1.4 | 0.0001 | 6.54 | 55017 | 1245 | 18.2 | 71 |
| Q768R5 | Plastidic 2-oxoglutarate/malate transporter | 1.4 | 0.0013 | 9.78 | 64031 | 69 | 4.5 | 4 |
| B4FAV3 | 40S ribosomal protein S3a | 1.39 | 0.0000 | 9.81 | 37453 | 243 | 44.2 | 13 |
| B4FGF1 | Non-green plastid inner envelope membrane protein | 1.39 | 0.0072 | 9.27 | 39021 | 226 | 11.7 | 7 |
| B4FQP1 | Putative oxidoreductase | 1.39 | 0.0224 | 9.2 | 40561 | 75 | 6.9 | 2 |
| B6TDR5 | Geranylgeranyl hydrogenase | 1.39 | 0.0001 | 9.15 | 57510 | 478 | 37.2 | 23 |
| C0HHN8 | Not assigned | 1.39 | 0.0018 | 8.91 | 29793 | 154 | 12.8 | 4 |
| C0PLI2 | Cold shock domain protein 1 | 1.39 | 0.0047 | 5.95 | 25500 | 467 | 33.7 | 14 |
| A0A096QLY8 | Nicotianamine synthase 3 | 1.38 | 0.0000 | 6.09 | 48502 | 48 | 9.7 | 4 |
| A0A096R5M3 | Cell division inhibitor-like | 1.38 | 0.0000 | 8.79 | 15270 | 55 | 17.7 | 1 |
| A0A096S5Z5 | Chlorophyll a-b binding protein 2 | 1.38 | 0.0000 | 8.35 | 37048 | 65 | 11.1 | 3 |
| A0A096UDB7 | Acetyl-CoA carboxylase 2 | 1.38 | 0.0009 | 5.89 | 287720 | 254 | 9.7 | 23 |
| C0PGA6 | Terpene cyclase/mutase family member | 1.38 | 0.0000 | 6.12 | 95708 | 35 | 4.1 | 1 |
| K7TW55 | Putative OEP86=outer envelope protein | 1.38 | 0.0000 | 4.4 | 163852 | 28 | 1.9 | 3 |
| P05641 | Photosystem II CP47 reaction center protein | 1.38 | 0.0000 | 6.06 | 59450 | 6027 | 31.5 | 226 |
| K7ULA1 | Putative calcium-dependent protein kinase family protein | 1.37 | 0.0014 | 5.79 | 70111 | 48 | 4.8 | 3 |
| K7VD90 | NADH dehydrogenase | 1.37 | 0.0001 | 7.59 | 65455 | 251 | 18.3 | 9 |
| K7VWJ6 | Chloroplast processing enzyme-like protein | 1.37 | 0.0015 | 5.98 | 153439 | 128 | 7.3 | 11 |
| Q41746 | Chlorophyll a/b-binding apoprotein CP26 | 1.37 | 0.0023 | 5.5 | 34620 | 1447 | 55.8 | 74 |
| A0A096PQC8 | Stem glycoprotein | 1.36 | 0.0054 | 8.68 | 34042 | 168 | 27.2 | 7 |
| A0A096QGR3 | Putative TCP-1/cpn60 chaperonin family protein isoform 1 | 1.36 | 0.0016 | 6.66 | 74252 | 71 | 6 | 3 |
| A0A096QNR3 | Not assigned | 1.36 | 0.0011 | 5.33 | 35467 | 154 | 17.2 | 8 |
| A0A096S2H5 | Not assigned | 1.36 | 0.0061 | 6.01 | 91145 | 944 | 29.4 | 32 |
| A0A096T4R6 | OSJNBb0079B02.1 protein | 1.36 | 0.0002 | 6.49 | 84563 | 147 | 7.8 | 5 |
| A0A096TS21 | Putative ABC protein | 1.36 | 0.0016 | 9.48 | 63538 | 93 | 14 | 6 |
| A0A096UH19 | Not assigned | 1.36 | 0.0005 | 4.96 | 40751 | 501 | 34.3 | 17 |
| B4FAB2 | Chaperone protein dnaJ | 1.36 | 0.0001 | 9.13 | 60039 | 363 | 21.1 | 20 |
| B4FAC5 | Not assigned | 1.36 | 0.0001 | 10.16 | 18862 | 126 | 21.1 | 6 |
| B4FHJ8 | Heterogeneous nuclear ribonucleoprotein A3 isoform 1 | 1.36 | 0.0000 | 5.97 | 43622 | 107 | 9.6 | 4 |
| B4FTA3 | 60S ribosomal protein L27 | 1.36 | 0.0000 | 10.64 | 20809 | 50 | 20.4 | 2 |
| B4FY31 | "DnaJ domain containing protein, expressed" | 1.36 | 0.0015 | 9.51 | 36271 | 144 | 15.5 | 5 |
| K7UPX8 | ABC-2 type transporter family protein | 1.36 | 0.0000 | 8.91 | 81425 | 45 | 3.1 | 1 |
| K7USR3 | Magnesium-protoporphyrin IX monomethyl ester [oxidative] cyclase | 1.36 | 0.0068 | 8.91 | 53835 | 399 | 35.8 | 18 |
| P02355 | "30S ribosomal protein S4, chloroplastic" | 1.36 | 0.0010 | 11.07 | 29005 | 106 | 27.9 | 6 |
| A0A096PYI9 | Chaperonin | 1.35 | 0.0000 | 9.34 | 11273 | 56 | 44.6 | 3 |
| A0A096QKN1 | OSJNBb0003B01.9 protein | 1.35 | 0.0013 | 6.22 | 39057 | 137 | 9.8 | 3 |
| A0A096RM67 | Chlorophyll a-b binding protein 8 | 1.35 | 0.0026 | 5.24 | 19091 | 135 | 16.5 | 4 |
| A0A096TR75 | Photosystem I assembly protein Ycf4 | 1.35 | 0.0123 | 9.85 | 23842 | 68 | 13 | 5 |
| B4FFZ7 | 60S ribosomal protein L32 | 1.35 | 0.0039 | 10.49 | 20283 | 91 | 22.6 | 4 |
| B4FVI0 | Aspartic proteinase nepenthesin-2 | 1.35 | 0.0000 | 6.23 | 51267 | 101 | 6.4 | 1 |
| B6SR73 | Tubulin alpha-6 chain | 1.35 | 0.0057 | 6.64 | 22631 | 69 | 6.1 | 2 |
| B6SZT9 | Chlorophyll a-b binding protein | 1.35 | 0.0054 | 5.24 | 30469 | 2855 | 61.2 | 127 |
| B6TB84 | Soluble inorganic pyrophosphatase | 1.35 | 0.0128 | 5.45 | 28033 | 166 | 30.8 | 9 |
| K7U8U5 | ABC1-like | 1.35 | 0.0032 | 5.53 | 93598 | 27 | 1.2 | 1 |
| O49010 | Herbicide safener binding protein | 1.35 | 0.0182 | 5.65 | 45209 | 71 | 13.2 | 3 |
| A0A096PFP7 | "Beta-glucosidase-like SFR2, chloroplastic" | 1.34 | 0.0000 | 5.84 | 80539 | 42 | 3.7 | 1 |
| A0A096PJM3 | Not assigned | 1.34 | 0.0144 | 8.11 | 43195 | 417 | 25.7 | 12 |
| A0A096SGZ5 | "EF hand family protein, expressed" | 1.34 | 0.0145 | 4.94 | 68624 | 59 | 6.9 | 3 |
| A0A0B4J312 | "Cell division cycle protein 48, putative, expressed" | 1.34 | 0.0035 | 5.06 | 103036 | 1043 | 39.4 | 43 |
| B4FB27 | Not assigned | 1.34 | 0.0004 | 8.85 | 47576 | 373 | 25.5 | 20 |
| B4FJN0 | RIP1 | 1.34 | 0.0006 | 5.77 | 47093 | 52 | 7.8 | 4 |
| C0PPF5 | Calcium sensing receptor | 1.34 | 0.0003 | 9.65 | 47661 | 601 | 49.9 | 35 |
| K7TTQ0 | Harpin inducing protein | 1.34 | 0.0038 | 9.7 | 41108 | 93 | 4.3 | 2 |
| K7W4U7 | Putative RAN GTPase activating family protein | 1.34 | 0.0058 | 8.03 | 75512 | 79 | 4.6 | 2 |
| A0A096R382 | "Alpha-1,4 glucan phosphorylase" | 1.33 | 0.0423 | 5.39 | 126422 | 182 | 7.6 | 5 |
| A0A096S0N9 | Salt stress root protein RS1 | 1.33 | 0.0018 | 4.82 | 37140 | 42 | 13.2 | 5 |
| A0A096TN76 | OSJNBb0011N17.20 protein | 1.33 | 0.0031 | 5.22 | 90679 | 163 | 7.4 | 3 |
| B4FCK9 | 60S ribosomal protein L22-2 | 1.33 | 0.0058 | 9.57 | 18905 | 335 | 39.2 | 12 |
| B4FHE4 | 60S ribosomal protein L17 | 1.33 | 0.0000 | 10.14 | 23848 | 73 | 35.7 | 10 |
| B4FR11 | Threonine endopeptidase | 1.33 | 0.0110 | 9.36 | 19374 | 193 | 19.1 | 5 |
| B6SJC8 | 60S ribosomal protein L18 | 1.33 | 0.0349 | 11.43 | 26228 | 243 | 32.6 | 10 |
| C0HH11 | Putative calcium-dependent protein kinase family protein isoform 1 | 1.33 | 0.0194 | 5.41 | 65111 | 68 | 8.5 | 2 |
| K7TMN5 | "AMP-binding enzyme family protein, expressed" | 1.33 | 0.0164 | 6.64 | 88279 | 83 | 8.5 | 7 |
| K7UZJ5 | Putative ABC transporter | 1.33 | 0.0436 | 6.83 | 91476 | 148 | 12.3 | 9 |
| K7VXL2 | Filamentation temperature-sensitive H 2A isoform 1 | 1.33 | 0.0004 | 5.68 | 80381 | 2516 | 47.1 | 111 |
| P48186 | "ATP synthase subunit b, chloroplastic" | 1.33 | 0.0001 | 9.27 | 24004 | 448 | 38.3 | 31 |
| A0A096Q440 | Putative UDP-glucosyltransferase | 1.32 | 0.0196 | 7.25 | 54348 | 56 | 3.8 | 3 |
| A0A096S1L7 | Tubulin beta-7 chain isoform 1 | 1.32 | 0.0000 | 5.05 | 29727 | 30 | 9.3 | 2 |
| A0A096T004 | Putative DEAD-box ATP-dependent RNA helicase family protein | 1.32 | 0.0000 | 9.65 | 43714 | 42 | 2.5 | 1 |
| A0A096TGG0 | Probable protein phosphatase 2C 5 | 1.32 | 0.0002 | 5.43 | 45265 | 263 | 21.4 | 9 |
| A0A096UBN6 | ABC1 family protein-like | 1.32 | 0.0200 | 9.81 | 39938 | 27 | 6.6 | 3 |
| A0A096UJK9 | "Chlorophyll A-B binding protein, expressed" | 1.32 | 0.0002 | 9.7 | 8838 | 281 | 27.9 | 12 |
| B4F957 | Pro-resilin | 1.32 | 0.0109 | 4.99 | 45656 | 363 | 24.1 | 12 |
| B4G0J5 | Histone deacetylase | 1.32 | 0.0000 | 5.43 | 56664 | 38 | 4.6 | 3 |
| B6UBZ9 | Cytochrome b-c1 complex subunit 7 | 1.32 | 0.0000 | 9.64 | 16396 | 125 | 37.3 | 6 |
| B7S825 | Lycopene epsilon cyclase1 | 1.32 | 0.0000 | 6.31 | 66372 | 44 | 2.2 | 1 |
| C0PDG8 | Putative signal peptide peptidase family protein | 1.32 | 0.0003 | 9.08 | 84862 | 441 | 16.4 | 8 |
| C4J3U5 | Putative Rickettsia surface antigen family protein | 1.32 | 0.0000 | 7.93 | 18556 | 74 | 14.9 | 1 |
| K7VN08 | ATP synthase B chain | 1.32 | 0.0001 | 5.48 | 26654 | 823 | 38.4 | 37 |
| A0A096QG13 | Expressed protein | 1.31 | 0.0000 | 5.06 | 15979 | 32 | 10.5 | 1 |
| A0A096QSZ4 | V-type proton ATPase subunit a | 1.31 | 0.0361 | 5.87 | 104660 | 197 | 9.2 | 6 |
| A0A096RBW7 | "Endomembrane protein 70 containing protein, expressed" | 1.31 | 0.0000 | 8.77 | 74328 | 38 | 1.5 | 1 |
| A0A096S2Q4 | "ATP-dependent zinc metalloprotease FTSH 1, chloroplastic" | 1.31 | 0.0005 | 5.62 | 81139 | 3149 | 47.7 | 109 |
| A0A096T303 | Putative cobW protein | 1.31 | 0.0006 | 8.61 | 92663 | 197 | 7.2 | 5 |
| A0A096TJ76 | Not assigned | 1.31 | 0.0017 | 9.72 | 43780 | 40 | 5.5 | 2 |
| B4FRV8 | OSJNBb0003B01.9 protein | 1.31 | 0.0040 | 8.89 | 37385 | 232 | 26 | 13 |
| B4FT80 | OB-fold nucleic acid binding domain containing protein | 1.31 | 0.0221 | 9.45 | 20368 | 135 | 16.9 | 1 |
| K7VGC0 | Vacuolar sorting receptor 7 | 1.31 | 0.0000 | 5.87 | 79048 | 28 | 1.3 | 1 |
| K7VPJ1 | Putative leucyl-tRNA synthetase | 1.31 | 0.0000 | 9.26 | 15595 | 60 | 9 | 1 |
| A0A096QEP1 | Serine hydroxymethyltransferase | 1.3 | 0.0075 | 7.63 | 66528 | 271 | 17.1 | 9 |
| A0A096QRL3 | "Cell elongation protein DIMINUTO, putative, expressed" | 1.3 | 0.0000 | 8.4 | 75724 | 23 | 3.4 | 1 |
| A0A096S532 | "KH domain containing protein, expressed" | 1.3 | 0.0008 | 5.28 | 58350 | 94 | 14.2 | 6 |
| B4FM95 | Not assigned | 1.3 | 0.0091 | 9.17 | 42180 | 89 | 5.2 | 3 |
| B6SIL9 | 40S ribosomal protein S27a | 1.3 | 0.0047 | 9.83 | 23873 | 637 | 46.5 | 36 |
| B6TVC7 | Ferredoxin | 1.3 | 0.0000 | 6.96 | 19199 | 70 | 7.3 | 1 |
| C0HGV6 | EST AU068209(C12438) corresponds to a region of the predicted gene | 1.3 | 0.0000 | 6.39 | 116009 | 156 | 8.4 | 6 |
| K7TFI4 | Not assigned | 1.3 | 0.0117 | 4.74 | 27786 | 289 | 27 | 19 |
| K7TZ83 | Putative sucrose-phosphate synthase family protein | 1.3 | 0.0015 | 6.65 | 124399 | 119 | 7.9 | 9 |
| K7U7W9 | "Magnesium-chelatase subunit ChlH, chloroplastic" | 1.3 | 0.0005 | 5.95 | 170889 | 228 | 7.8 | 10 |
| Q94IQ8 | Putative methyl-binding domain protein MBD106 | 1.3 | 0.0012 | 4.4 | 51527 | 90 | 5.4 | 3 |
| **Down-regulation** |  |  |  |  |  |  |  |  |
| B6T5U0 | F1F0-ATPase inhibitor protein | 0.4 | 0.0136 | 9.52 | 15733 | 109 | 20.3 | 3 |
| A0A096RTN1 | Pathogenesis-related protein 10b | 0.48 | 0.0001 | 5.36 | 19581 | 129 | 30 | 4 |
| B4FA32 | Peroxidase | 0.5 | 0.0002 | 6.49 | 35933 | 92 | 9.6 | 3 |
| Q9FQA5 | Glutathione S-transferase GST 34 | 0.52 | 0.0000 | 5.63 | 28052 | 70 | 8.9 | 1 |
| B4FV91 | Thaumatin-like protein | 0.54 | 0.0113 | 4.59 | 18522 | 70 | 20.1 | 2 |
| K7VLR3 | Putative O-Glycosyl hydrolase superfamily protein | 0.59 | 0.0000 | 4.7 | 38221 | 26 | 2.4 | 1 |
| B6SQM0 | Major pollen allergen Car b 1 isoforms 1A and 1B | 0.6 | 0.0183 | 4.99 | 19752 | 51 | 16.2 | 1 |
| A0A096QXN0 | "Putative beta-1,3-glucanase" | 0.61 | 0.0055 | 4.39 | 36087 | 81 | 9 | 2 |
| A0A096QEZ3 | Putative receptor serine/threonine kinase PR5K | 0.63 | 0.0008 | 4.13 | 26303 | 269 | 23 | 5 |
| K7VA33 | Not assigned | 0.64 | 0.0009 | 6.15 | 11393 | 97 | 40.4 | 7 |
| B4FN73 | 2-oxoglutarate-dependent dioxygenase DAO | 0.65 | 0.0000 | 5.32 | 34769 | 69 | 11.7 | 4 |
| C4J5Y0 | Protein binding protein | 0.65 | 0.0014 | 4.82 | 62883 | 414 | 30.8 | 21 |
| B4FVB1 | Actin-3 | 0.66 | 0.0382 | 5.23 | 46740 | 1083 | 46.4 | 68 |
| B6U579 | Thaumatin-like protein | 0.66 | 0.0006 | 5.84 | 23820 | 64 | 13.2 | 4 |
| A0A096PNZ3 | CHY1 | 0.67 | 0.0000 | 6.23 | 55680 | 31 | 3.1 | 1 |
| A0A096RTH2 | 5-methyltetrahydropteroyltriglutamate--homocysteine methyltransferase 2 | 0.67 | 0.0090 | 6.38 | 101927 | 405 | 16.7 | 21 |
| A0A096UC41 | 2-hydroxy-3-oxopropionate reductase | 0.67 | 0.0019 | 8.24 | 40530 | 76 | 7 | 3 |
| B4G264 | Putative glucosyltransferase-2 | 0.68 | 0.0006 | 5.8 | 54221 | 272 | 17.9 | 11 |
| C0HI97 | Not assigned | 0.68 | 0.0028 | 5.72 | 23806 | 82 | 22.7 | 5 |
| Q9FQA9 | Glutathione S-transferase GST 30 | 0.68 | 0.0068 | 5.33 | 27385 | 202 | 21.6 | 9 |
| A0A096R6E1 | Not assigned | 0.69 | 0.0003 | 5.18 | 19422 | 31 | 17.9 | 3 |
| A0A096RJI8 | Blight-associated protein p12 | 0.69 | 0.0282 | 4.19 | 14479 | 39 | 6.1 | 2 |
| B4FPG3 | Splicing factor 3A subunit 2 | 0.69 | 0.0000 | 9.77 | 40951 | 29 | 2.5 | 1 |
| Q6VWJ0 | Caffeoyl-CoA 3-O-methyltransferase 1 | 0.69 | 0.0000 | 5.14 | 33155 | 38 | 8.9 | 2 |
| A0A096SFK1 | DNA binding protein PF1 | 0.7 | 0.0000 | 9.34 | 36224 | 29 | 4.9 | 2 |
| B4FVP5 | Pathogeneis protein1 | 0.7 | 0.0158 | 4.38 | 18127 | 216 | 37.4 | 6 |
| B6TLX6 | PYM protein | 0.7 | 0.0000 | 9.62 | 31406 | 48 | 8.8 | 2 |
| B6TUZ6 | Macrophage migration inhibitory factor | 0.7 | 0.0009 | 5.73 | 13433 | 169 | 14.8 | 5 |
| C0PDJ5 | Indole-3-acetate beta-glucosyltransferase | 0.7 | 0.0010 | 5.29 | 55120 | 117 | 6.3 | 3 |
| P33679 | Zeamatin | 0.7 | 0.0012 | 7.84 | 26729 | 410 | 39.6 | 19 |
| A0A096UFQ8 | Ribose-5-phosphate isomerase | 0.71 | 0.0321 | 5.44 | 35376 | 110 | 7 | 2 |
| B4FR89 | Phosphomannomutase | 0.71 | 0.0136 | 5.83 | 32179 | 84 | 14.5 | 2 |
| B6U9Z8 | Glutathione S-transferase GSTU6 | 0.71 | 0.0006 | 5.71 | 28242 | 227 | 28.9 | 8 |
| A0A096R3M8 | Phenylalanine ammonia-lyase | 0.72 | 0.0000 | 6.07 | 68326 | 46 | 5.7 | 4 |
| B4FDT9 | Small nuclear ribonucleoprotein Sm D2 | 0.72 | 0.0000 | 9.95 | 15376 | 76 | 35.2 | 4 |
| B6SX84 | Plastid-specific ribosomal protein 6 | 0.72 | 0.0000 | 9.73 | 14593 | 39 | 6.6 | 2 |
| B6SZK3 | NAD(P)H-dependent oxidoreductase | 0.72 | 0.0014 | 5.69 | 42605 | 307 | 42.5 | 13 |
| B6TDH3 | Expressed protein | 0.72 | 0.0001 | 9.36 | 21418 | 98 | 14.8 | 2 |
| B8A0E5 | "Hydrolase, carbon-nitrogen family protein, expressed" | 0.72 | 0.0015 | 7.08 | 41977 | 202 | 18.5 | 9 |
| K7TSQ4 | Carboxypeptidase | 0.72 | 0.0000 | 6.22 | 64379 | 185 | 8.1 | 4 |
| A0A096UJE7 | Putative UDP-glucose pyrophosphorylase | 0.73 | 0.0008 | 6.64 | 29670 | 1126 | 76.4 | 63 |
| B4FI06 | MBF1 transcription factor | 0.73 | 0.0000 | 9.99 | 20236 | 149 | 36.6 | 6 |
| B6TL20 | Glutathione S-transferase GSTU6 | 0.73 | 0.0025 | 5.81 | 28581 | 76 | 17.7 | 4 |
| K7TTR2 | Not assigned | 0.73 | 0.0000 | 7.85 | 15381 | 260 | 25.4 | 7 |
| K7VC35 | S-adenosylmethionine synthase | 0.73 | 0.0000 | 5.5 | 49157 | 266 | 24.5 | 14 |
| K7VPT6 | Not assigned | 0.73 | 0.0003 | 10.08 | 18417 | 215 | 15.1 | 6 |
| P04907 | Glutathione S-transferase 3 | 0.73 | 0.0000 | 6.05 | 26412 | 941 | 57.7 | 35 |
| A0A096RE18 | Putative inorganic pyrophosphatase | 0.74 | 0.0006 | 9.6 | 18636 | 440 | 53.1 | 36 |
| A0A096S5S4 | Helminthosporium carbonum susceptibility2 | 0.74 | 0.0002 | 5.16 | 39386 | 128 | 14.5 | 6 |
| A0A096T1S3 | Nucleoside diphosphate kinase | 0.74 | 0.0037 | 5.65 | 20667 | 172 | 30.8 | 9 |
| B4FN58 | Protein-L-isoaspartate O-methyltransferase | 0.74 | 0.0030 | 5.57 | 27631 | 87 | 15.9 | 2 |
| B4G1B0 | Remorin | 0.74 | 0.0006 | 5.74 | 29667 | 83 | 26.6 | 9 |
| B6SK54 | Superoxide dismutase [Cu-Zn] | 0.74 | 0.0091 | 5.64 | 16780 | 418 | 27.6 | 16 |
| B6T969 | Proteasome subunit alpha type | 0.74 | 0.0461 | 5.53 | 29531 | 346 | 34.9 | 14 |
| B6TM36 | Not assigned | 0.74 | 0.0000 | 10.03 | 22071 | 43 | 7.8 | 2 |
| C0HGH7 | Not assigned | 0.74 | 0.0099 | 5.87 | 23555 | 82 | 14.8 | 5 |
| C0PMH6 | Glucose-6-phosphate/phosphate translocator 2 Glucose-6-phosphate/phosphate-translocator | 0.74 | 0.0000 | 9.68 | 48088 | 34 | 5.1 | 1 |
| K7U0Q4 | Germin-like protein subfamily 1 member 11 | 0.74 | 0.0016 | 7.77 | 28567 | 68 | 4.8 | 2 |
| K7VCB9 | Hydroxyethylthiazole kinase | 0.74 | 0.0000 | 5.6 | 31299 | 39 | 4.3 | 1 |
| A0A096Q1T0 | Cytochrome b | 0.75 | 0.0000 | 7.83 | 46513 | 61 | 2.3 | 2 |
| A0A096RLQ7 | Ubiquitin-conjugating enzyme E2 5B | 0.75 | 0.0015 | 6.4 | 18699 | 70 | 7.7 | 2 |
| A0A096SCI0 | Not assigned | 0.75 | 0.0003 | 4.93 | 52368 | 172 | 17.9 | 5 |
| A0A096SMI4 | Putative ubiquitin-conjugating enzyme family | 0.75 | 0.0000 | 7.77 | 15804 | 40 | 9.2 | 1 |
| A0A096T3A1 | Neutral ceramidase | 0.75 | 0.0005 | 6.37 | 95542 | 132 | 4.8 | 4 |
| B4FCW3 | SAM domain protein | 0.75 | 0.0000 | 10.09 | 28633 | 32 | 4.7 | 1 |
| B4FES7 | "Hsp20/alpha crystallin family protein, expressed" | 0.75 | 0.0027 | 9.3 | 26604 | 142 | 14.9 | 5 |
| B4FHA0 | Not assigned | 0.75 | 0.0008 | 7.44 | 27131 | 948 | 45.3 | 52 |
| B6SIF0 | Glycine-rich RNA-binding protein 2 | 0.75 | 0.0016 | 6.58 | 17130 | 278 | 30.8 | 11 |
| B6SSB9 | Plastocyanin | 0.75 | 0.0000 | 5.62 | 17293 | 724 | 20.6 | 40 |
| C0PCL6 | CHCH domain containing protein | 0.75 | 0.0000 | 9.22 | 26312 | 29 | 8.8 | 1 |
| K7UND8 | Not assigned | 0.75 | 0.0095 | 7.77 | 28055 | 273 | 19.3 | 8 |
| K7V7L8 | Not assigned | 0.75 | 0.0154 | 8.57 | 29704 | 83 | 6.9 | 3 |
| A0A096U686 | Oxygen-evolving enhancer protein 3-1 | 0.76 | 0.0045 | 9.8 | 27392 | 1421 | 48.6 | 96 |
| B4F9J1 | Beta-galactosidase | 0.76 | 0.0000 | 8.13 | 87000 | 37 | 2.4 | 1 |
| B4FGM4 | Endothelial differentiation-related factor 1 | 0.76 | 0.0078 | 9.95 | 20222 | 155 | 30.3 | 7 |
| B6T4K8 | Expressed protein | 0.76 | 0.0039 | 5.22 | 20615 | 235 | 26.3 | 4 |
| B6T6D7 | MIR-interacting saposin-like protein | 0.76 | 0.0000 | 5.54 | 25051 | 29 | 11.8 | 2 |
| B6TFF1 | Putative thioredoxin superfamily protein | 0.76 | 0.0016 | 9.54 | 22002 | 99 | 15.8 | 5 |
| B7ZZ04 | "Jasmonate-induced protein, putative, expressed" | 0.76 | 0.0017 | 5.96 | 34622 | 146 | 6.5 | 2 |
| B8A043 | Transcribed sequence 1087 protein | 0.76 | 0.0088 | 5.76 | 75169 | 130 | 11.3 | 9 |
| B8A0A6 | "26S protease regulatory subunit 4, putative, expressed" | 0.76 | 0.0127 | 6.05 | 58755 | 305 | 34.8 | 14 |
| C0PFN4 | Apospory-associated protein isoform 1 | 0.76 | 0.0000 | 6.26 | 42415 | 318 | 23.2 | 13 |
| C0PH53 | Cat eye syndrome critical region protein 5 | 0.76 | 0.0000 | 7.82 | 47838 | 43 | 7.8 | 1 |
| K7TKE6 | 40S ribosomal protein S10 | 0.76 | 0.0032 | 9.83 | 22668 | 204 | 42.2 | 14 |
| K7VH58 | Peroxidase | 0.76 | 0.0001 | 8.59 | 34177 | 632 | 59.7 | 26 |
| Q41864 | "Thioredoxin M-type, chloroplastic" | 0.76 | 0.0021 | 8.7 | 21440 | 1454 | 43.1 | 63 |
| Q6VWG3 | Brown midrib3 | 0.76 | 0.0007 | 5.49 | 44224 | 302 | 34.6 | 14 |
| A0A096RY69 | "Glutathione S-transferase, N-terminal domain containing protein, expressed" | 0.77 | 0.0004 | 7.19 | 41345 | 422 | 24 | 19 |
| A0A096T020 | "Harpin-induced protein 1 containing protein, expressed" | 0.77 | 0.0000 | 9.1 | 25000 | 58 | 12.3 | 1 |
| A0A096T686 | Peroxidase | 0.77 | 0.0015 | 5.25 | 37573 | 62 | 7.1 | 3 |
| A0A096TRV6 | "Putative beta-1,3-glucanase" | 0.77 | 0.0042 | 6.84 | 74823 | 302 | 14.8 | 7 |
| A0A0B4J3G7 | Peroxidase | 0.77 | 0.0106 | 6.18 | 36053 | 207 | 28.8 | 8 |
| B4F9M6 | Transcribed sequence 1087 protein | 0.77 | 0.0024 | 6.01 | 74696 | 112 | 15.8 | 9 |
| B4FBW5 | Mannitol dehydrogenase | 0.77 | 0.0217 | 6.82 | 44195 | 61 | 5.6 | 3 |
| B4FQP4 | Probable 4-coumarate--CoA ligase 3 | 0.77 | 0.0011 | 5.2 | 67404 | 114 | 8.5 | 7 |
| B4G195 | Aspartate aminotransferase | 0.77 | 0.0001 | 8.39 | 54660 | 143 | 12.4 | 7 |
| B4G1T3 | Acidic class III chitinase OsChib3a | 0.77 | 0.0161 | 4.06 | 31992 | 224 | 8.8 | 8 |
| B6SRG3 | Not assigned | 0.77 | 0.0342 | 10.38 | 16248 | 32 | 17.9 | 2 |
| B6T1R4 | OB-fold nucleic acid binding domain containing protein | 0.77 | 0.0030 | 6.75 | 17246 | 74 | 19.7 | 3 |

^a^ Protein accession number of UniProt database used for search.

^b^Protein functional description.

^c^ Protein molecular weight.

^d^ Amino acid sequence coverage.
