# Supplementary material for: Physiological and proteome studies of maize (Zea mays L.) in response to leaf removal under high plant density
Source: BMC Plant Biol. 2018 Dec 29;18:378. doi: 10.1186/s12870-018-1607-8 (PMC6310946; doi:10.1186/s12870-018-1607-8)
Supplement: Supplementary file 4 — Table S3. Differences in protein abundances between the four-leaf removal treatment (S4) and the control (S0). (DOCX 48 kb) [file 12870_2018_1607_MOESM4_ESM.docx]

**Table S3.** Differences in protein abundances between the two-leaf removal treatment (S_4_) and the control (S_0_).

| **Protein** | **Protein description^b^** | **Ratio** | **P** | **pI** | **MW^c^** | **Score** | **AASC^d^** | **Number of** |
| --- | --- | --- | --- | --- | --- | --- | --- | --- |
| **accession^a^** |  | **S_4_/S_0_** | **value** |  | **[kDa]** |  | **[%]** | **peptide** |
| **Up-regulation** |  |  |  |  |  |  |  |  |
| B4FV78 | Acyl carrier protein | 1.7 | 0.0100 | 5.24 | 17616 | 150 | 16.3 | 3 |
| A0A096RX02 | Calmodulin1 | 1.66 | 0.0004 | 4.57 | 25519 | 278 | 16.7 | 6 |
| B6U3H3 | CP12-1 | 1.65 | 0.0029 | 4.73 | 16777 | 128 | 40.7 | 5 |
| K7UBT7 | "HEAT repeat, putative" | 1.61 | 0.0000 | 8.29 | 176673 | 49 | 1.7 | 1 |
| A0A096PIP9 | "DEAD-box ATP-dependent RNA helicase 3, chloroplastic" | 1.56 | 0.0015 | 6.51 | 87625 | 200 | 16 | 5 |
| A0A096RZ51 | "KH domain-containing protein, putative, expressed" | 1.55 | 0.0211 | 7.38 | 92017 | 40 | 1.2 | 2 |
| A0A096TL89 | Not assigned | 1.54 | 0.0000 | 13.01 | 31845 | 46 | 4.5 | 2 |
| C0P456 | OSJNBb0038F03.9 protein | 1.54 | 0.0363 | 7.04 | 67900 | 58 | 3.9 | 1 |
| C0PLI2 | Cold shock domain protein 1 | 1.54 | 0.0031 | 5.95 | 25500 | 467 | 33.7 | 14 |
| K7TL05 | 14-3-3-like protein GF14-12 | 1.54 | 0.0002 | 4.75 | 34079 | 881 | 65.5 | 43 |
| A0A096TN17 | Putative ubiquitin-protein ligase 1 | 1.52 | 0.0000 | 5.13 | 438899 | 38 | 1 | 2 |
| C4J3E2 | OSJNBb0028M18.1 protein | 1.52 | 0.0013 | 5.07 | 91605 | 93 | 14.9 | 6 |
| B4FSN2 | Inner membrane protein ALBINO3 | 1.51 | 0.0077 | 9.3 | 54780 | 118 | 7 | 6 |
| B4FRE3 | Ankyrin repeat domain-containing protein 2 | 1.49 | 0.0149 | 4.57 | 43244 | 139 | 17.5 | 8 |
| A0A096QBR2 | Sucrose transport protein SUT1 | 1.48 | 0.0000 | 8.76 | 60705 | 31 | 3.8 | 1 |
| A0A096TGH7 | von Willebrand factor type A domain containing protein | 1.48 | 0.0039 | 6.88 | 74586 | 114 | 12.5 | 7 |
| B4FQN6 | Sorbitol transporter | 1.46 | 0.0000 | 7.71 | 59606 | 29 | 3.4 | 2 |
| B6TP60 | CSD transcription factor | 1.46 | 0.0001 | 5.92 | 22180 | 142 | 24.5 | 4 |
| A0A096R9R5 | Glycine-rich RNA-binding protein-like | 1.45 | 0.0000 | 9.24 | 29518 | 27 | 3.9 | 1 |
| A0A096UDG8 | "50S ribosomal protein L32, chloroplastic" | 1.44 | 0.0000 | 10.41 | 8915 | 29 | 15.5 | 2 |
| C4J6C7 | Eukaryotic translation initiation factor 1A | 1.44 | 0.0000 | 5.08 | 20173 | 51 | 14.6 | 2 |
| A0A096U8E3 | Putative TGB12K interacting protein 3 | 1.43 | 0.0000 | 4.64 | 35820 | 115 | 15.5 | 5 |
| B4FTL3 | Putative cinnamoyl CoA reductase | 1.43 | 0.0004 | 6.2 | 44711 | 73 | 9.4 | 4 |
| C0HGV6 | EST AU068209(C12438) corresponds to a region of the predicted gene | 1.43 | 0.0000 | 6.39 | 116009 | 156 | 8.4 | 7 |
| A0A096QGR3 | Putative TCP-1/cpn60 chaperonin family protein isoform 1 | 1.41 | 0.0039 | 6.66 | 74252 | 71 | 6 | 3 |
| A0A096QPQ8 | Not assigned | 1.4 | 0.0000 | 6.4 | 24156 | 23 | 6.1 | 1 |
| A0A096PWM9 | Ubiquinol oxidase | 1.39 | 0.0000 | 9.22 | 67940 | 39 | 3.8 | 2 |
| A0A096R3L2 | Cellulose synthase-like protein H2 | 1.39 | 0.0000 | 9.03 | 44357 | 66 | 6.6 | 2 |
| A0A096SF87 | Not assigned | 1.39 | 0.0000 | 5.23 | 20863 | 144 | 26.4 | 6 |
| A0A096UDB7 | Acetyl-CoA carboxylase 2 | 1.39 | 0.0003 | 5.89 | 287720 | 254 | 9.7 | 23 |
| A0A096T004 | Putative DEAD-box ATP-dependent RNA helicase family protein | 1.38 | 0.0000 | 9.65 | 43714 | 42 | 2.5 | 1 |
| K7UQU1 | Putative glucosyltransferase | 1.38 | 0.0043 | 5.11 | 56513 | 168 | 9.4 | 3 |
| P00835 | "ATP synthase epsilon chain, chloroplastic" | 1.38 | 0.0184 | 5.03 | 16869 | 366 | 20.4 | 20 |
| A0A096RTE0 | Aldo/keto reductase family-like protein | 1.37 | 0.0151 | 8.11 | 46407 | 27 | 2.6 | 1 |
| B1P759 | Chloroplast ferredoxin 1 | 1.37 | 0.0000 | 4.59 | 17069 | 240 | 34.7 | 5 |
| Q5EUD5 | Protein disulfide isomerase | 1.37 | 0.0000 | 5.45 | 56203 | 35 | 4.6 | 2 |
| A0A096Q176 | Putative steroid 22-alpha-hydroxylase | 1.36 | 0.0020 | 8.8 | 75901 | 208 | 8.9 | 5 |
| B4FWQ8 | DCL protein-like | 1.36 | 0.0000 | 6.27 | 29037 | 65 | 11.9 | 2 |
| C0P699 | Elongation factor Tu | 1.36 | 0.0007 | 6.2 | 57160 | 1961 | 45.5 | 76 |
| A0A096R0X1 | T-complex protein 1 subunit delta | 1.35 | 0.0000 | 5.59 | 69443 | 334 | 28.9 | 17 |
| A0A096TGY8 | Not assigned | 1.35 | 0.0000 | 5.96 | 120969 | 28 | 1.8 | 1 |
| C0PCS9 | AMP-binding protein | 1.35 | 0.0000 | 7.49 | 46790 | 102 | 10.3 | 3 |
| B6SR73 | Tubulin alpha-6 chain | 1.34 | 0.0112 | 6.64 | 22631 | 69 | 6.1 | 2 |
| P49085 | "Phytoene synthase, chloroplastic" | 1.34 | 0.0000 | 8.81 | 51205 | 51 | 4.4 | 1 |
| A0A096R2J5 | OSJNBb0108J11.6 protein | 1.33 | 0.0000 | 8.12 | 49366 | 41 | 2 | 1 |
| A0A096RLN4 | Ubiquinol oxidase | 1.33 | 0.0000 | 5.52 | 17159 | 42 | 7.9 | 2 |
| A0A096S1S1 | Expressed protein | 1.33 | 0.0000 | 5.56 | 71316 | 41 | 4.8 | 2 |
| A0A096T026 | Not assigned | 1.33 | 0.0000 | 9.43 | 37557 | 33 | 2.6 | 1 |
| B4FA19 | Putative L-cysteine desulfhydrase 1 | 1.33 | 0.0006 | 5.98 | 55644 | 130 | 12 | 6 |
| B4FT61 | Not assigned | 1.33 | 0.0008 | 6.11 | 38222 | 37 | 6.3 | 2 |
| B6U284 | 14-3-3-like protein | 1.33 | 0.0127 | 4.78 | 33773 | 441 | 57.4 | 26 |
| C0HFG8 | 3-ketoacyl-CoA synthase | 1.33 | 0.0000 | 9.19 | 65926 | 36 | 2.9 | 2 |
| G3K3T1 | GTP-binding protein TypA | 1.33 | 0.0000 | 6.8 | 82736 | 267 | 16.4 | 10 |
| K7U8U5 | ABC1-like | 1.33 | 0.0327 | 5.53 | 93598 | 27 | 1.2 | 1 |
| A0A096SID7 | 9-cis-epoxycarotenoid dioxygenase 1 | 1.32 | 0.0047 | 8.64 | 83173 | 110 | 7.4 | 6 |
| B4FAC5 | Not assigned | 1.32 | 0.0007 | 10.16 | 18862 | 126 | 21.1 | 6 |
| B4FNZ2 | Tubulin-specific chaperone A | 1.32 | 0.0022 | 5.08 | 16053 | 60 | 48.2 | 3 |
| K7TQX2 | Not assigned | 1.32 | 0.0013 | 4.68 | 66267 | 232 | 20.4 | 10 |
| P24993 | Photosystem II reaction center protein H | 1.32 | 0.0001 | 8.09 | 8928 | 212 | 16.4 | 13 |
| A0A096Q2B6 | Probable cytokinin riboside 5'-monophosphate phosphoribohydrolase LOGL9 | 1.31 | 0.0037 | 9.73 | 24559 | 50 | 9 | 2 |
| A0A096Q440 | Putative UDP-glucosyltransferase | 1.31 | 0.0156 | 7.25 | 54348 | 56 | 3.8 | 3 |
| A0A096RHG9 | "Probable DNA gyrase subunit A, chloroplastic/mitochondrial" | 1.31 | 0.0072 | 8.91 | 114055 | 40 | 3.1 | 2 |
| A0A096U038 | Cytochrome c oxidase subunit Vb | 1.31 | 0.0298 | 5.11 | 18196 | 48 | 8.3 | 2 |
| A0A096UHG3 | Not assigned | 1.31 | 0.0000 | 5.48 | 80373 | 479 | 32.3 | 21 |
| B6T927 | Not assigned | 1.31 | 0.0001 | 5.02 | 28430 | 268 | 41.3 | 18 |
| C0P9K3 | Inner membrane protein ALBINO3 | 1.31 | 0.0000 | 8.92 | 36965 | 81 | 8.4 | 6 |
| K7TZ83 | Putative sucrose-phosphate synthase family protein | 1.31 | 0.0008 | 6.65 | 124399 | 119 | 7.9 | 9 |
| A0A096QUG5 | "NADH-dependent oxidoreductase 1, putative, expressed" | 1.3 | 0.0000 | 5.93 | 32238 | 73 | 10 | 2 |
| A0A096SEN0 | Splicing factor 45 | 1.3 | 0.0000 | 9.63 | 35782 | 63 | 3.8 | 1 |
| A0A096T780 | Putative tetratricopeptide repeat(TPR)-containing protein | 1.3 | 0.0078 | 5.75 | 220658 | 188 | 8.6 | 14 |
| K7VI25 | "ATPase 3, putative, expressed" | 1.3 | 0.0019 | 6.23 | 66659 | 123 | 10.4 | 4 |
| **Down-regulation** |  |  |  |  |  |  |  |  |
| A0A096PQC8 | Stem glycoprotein | 0.52 | 0.0054 | 8.68 | 34042 | 168 | 27.2 | 7 |
| A0A096QXN0 | "Putative beta-1,3-glucanase" | 0.52 | 0.0107 | 4.39 | 36087 | 81 | 9 | 2 |
| A0A096RTN1 | Pathogenesis-related protein 10b | 0.53 | 0.0014 | 5.36 | 19581 | 129 | 30 | 4 |
| Q9FQA5 | Glutathione S-transferase GST 34 | 0.53 | 0.0000 | 5.63 | 28052 | 70 | 8.9 | 1 |
| B4FA32 | Peroxidase | 0.54 | 0.0006 | 6.49 | 35933 | 92 | 9.6 | 3 |
| B4FV91 | Thaumatin-like protein | 0.54 | 0.0053 | 4.59 | 18522 | 70 | 20.1 | 2 |
| A0A096QEZ3 | Putative receptor serine/threonine kinase PR5K | 0.59 | 0.0016 | 4.13 | 26303 | 269 | 23 | 5 |
| K7TJS0 | OSJNBb0062H02.3 protein | 0.6 | 0.0412 | 5.95 | 49183 | 140 | 8.5 | 2 |
| A0A096RJI8 | Blight-associated protein p12 | 0.63 | 0.0139 | 4.19 | 14479 | 39 | 6.1 | 2 |
| K7TSQ4 | Carboxypeptidase | 0.63 | 0.0000 | 6.22 | 64379 | 185 | 8.1 | 4 |
| A0A096SNU5 | Putative RNase S-like protein | 0.66 | 0.0001 | 8.58 | 33640 | 411 | 35.4 | 20 |
| B4FVP5 | Pathogeneis protein1 | 0.66 | 0.0183 | 4.38 | 18127 | 216 | 37.4 | 6 |
| A0A096R0L1 | Light-regulated protein | 0.67 | 0.0174 | 8.74 | 12053 | 148 | 24.1 | 13 |
| B4FTR1 | Undecaprenyl pyrophosphate synthetase | 0.67 | 0.0019 | 9.81 | 35549 | 52 | 10.1 | 2 |
| B4G0H7 | Not assigned | 0.68 | 0.0002 | 7.85 | 28175 | 220 | 29.3 | 8 |
| K7V5L0 | UMP synthase | 0.7 | 0.0000 | 8.24 | 133447 | 91 | 3.2 | 7 |
| B4FHK4 | Not assigned | 0.71 | 0.0009 | 6.53 | 48707 | 761 | 47.9 | 43 |
| B4FN76 | Light-regulated protein | 0.71 | 0.0000 | 4.93 | 15532 | 124 | 22.5 | 12 |
| P33679 | Zeamatin | 0.71 | 0.0001 | 7.84 | 26729 | 410 | 39.6 | 19 |
| Q42420 | Proteinase inhibitor | 0.71 | 0.0001 | 5.63 | 9032 | 109 | 71.2 | 3 |
| A0A096R6E1 | Not assigned | 0.72 | 0.0048 | 5.18 | 19422 | 31 | 17.9 | 3 |
| A0A096RTH2 | 5-methyltetrahydropteroyltriglutamate--homocysteine methyltransferase 2 | 0.72 | 0.0106 | 6.38 | 101927 | 405 | 16.7 | 21 |
| A0A096QR92 | Not assigned | 0.73 | 0.0224 | 5.68 | 48475 | 135 | 22.6 | 9 |
| A0A096RGK0 | Germin-like protein 4-1 | 0.73 | 0.0037 | 7.79 | 28598 | 123 | 15.2 | 6 |
| A0A096RR57 | Oryzain gamma chain | 0.73 | 0.0078 | 5.21 | 26972 | 136 | 26 | 5 |
| A8IK79 | Asr protein | 0.73 | 0.0005 | 6.64 | 15449 | 136 | 25 | 2 |
| B4G015 | "Thiamine thiazole synthase, chloroplastic" | 0.73 | 0.0013 | 5.59 | 40205 | 479 | 24.8 | 12 |
| B6U783 | MYB-related transcription factor | 0.73 | 0.0057 | 5.35 | 10468 | 76 | 42.3 | 2 |
| A0A096UFQ8 | Ribose-5-phosphate isomerase | 0.74 | 0.0037 | 5.44 | 35376 | 110 | 7 | 2 |
| A0A0B4J3G7 | Peroxidase | 0.74 | 0.0066 | 6.18 | 36053 | 207 | 28.8 | 8 |
| B4FQP4 | Probable 4-coumarate--CoA ligase 3 | 0.74 | 0.0062 | 5.2 | 67404 | 114 | 8.5 | 7 |
| B4G1T3 | Acidic class III chitinase OsChib3a | 0.74 | 0.0062 | 4.06 | 31992 | 224 | 8.8 | 8 |
| B4FTG2 | 3-methyl-2-oxobutanoate hydroxymethyltransferase | 0.76 | 0.0000 | 7.67 | 41058 | 90 | 4.3 | 1 |
| B4G1V7 | Expressed protein | 0.76 | 0.0025 | 4.73 | 20024 | 384 | 63 | 23 |
| B6SQM0 | Major pollen allergen Car b 1 isoforms 1A and 1B | 0.76 | 0.0424 | 4.99 | 19752 | 51 | 16.2 | 1 |
| B6UET1 | Nicotinate phosphoribosyltransferase-like protein | 0.76 | 0.0000 | 6.89 | 70951 | 69 | 5.5 | 2 |
| K7TGW6 | Putative polyphenol oxidase family protein | 0.76 | 0.0001 | 8.09 | 76932 | 475 | 22.8 | 18 |
| A0A096Q1Q9 | Expressed protein | 0.77 | 0.0000 | 9.86 | 28058 | 37 | 3.3 | 1 |
| B4FCW3 | SAM domain protein | 0.77 | 0.0000 | 10.09 | 28633 | 32 | 4.7 | 1 |
| C0PH53 | Cat eye syndrome critical region protein 5 | 0.77 | 0.0000 | 7.82 | 47838 | 43 | 7.8 | 1 |
| C4JA36 | Putative RNA recognition motif containing family protein | 0.77 | 0.0001 | 6.6 | 33781 | 110 | 11.2 | 4 |
| K7VLR3 | Putative O-Glycosyl hydrolase superfamily protein | 0.77 | 0.0000 | 4.7 | 38221 | 26 | 2.4 | 1 |

^a^ Protein accession number of UniProt database used for search.

^b^Protein functional description.

^c^ Protein molecular weight.

^d^ Amino acid sequence coverage.
