# Supplementary material for: Physiological and proteome studies of maize (Zea mays L.) in response to leaf removal under high plant density
Source: BMC Plant Biol. 2018 Dec 29;18:378. doi: 10.1186/s12870-018-1607-8 (PMC6310946; doi:10.1186/s12870-018-1607-8)
Supplement: Supplementary file 5 — Figure S2. Gene ontology (GO) classification of differentially accumulated proteins. (A) Up-regulated proteins with S2 treatment compared to S0 treatment; (B) down-regulated proteins with S2 treatment compared to S0 treatment; (C) up-regulated proteins with S4 treatment compared to S0 treatment; and (D) down-regulated proteins with S4 treatment compared to S0 treatment. S0 refers to control (no leaf removal); S2 and S4 refer to the removal of two and four uppermost leaves, respectively. (PDF 199 kb) [file 12870_2018_1607_MOESM5_ESM.pdf]

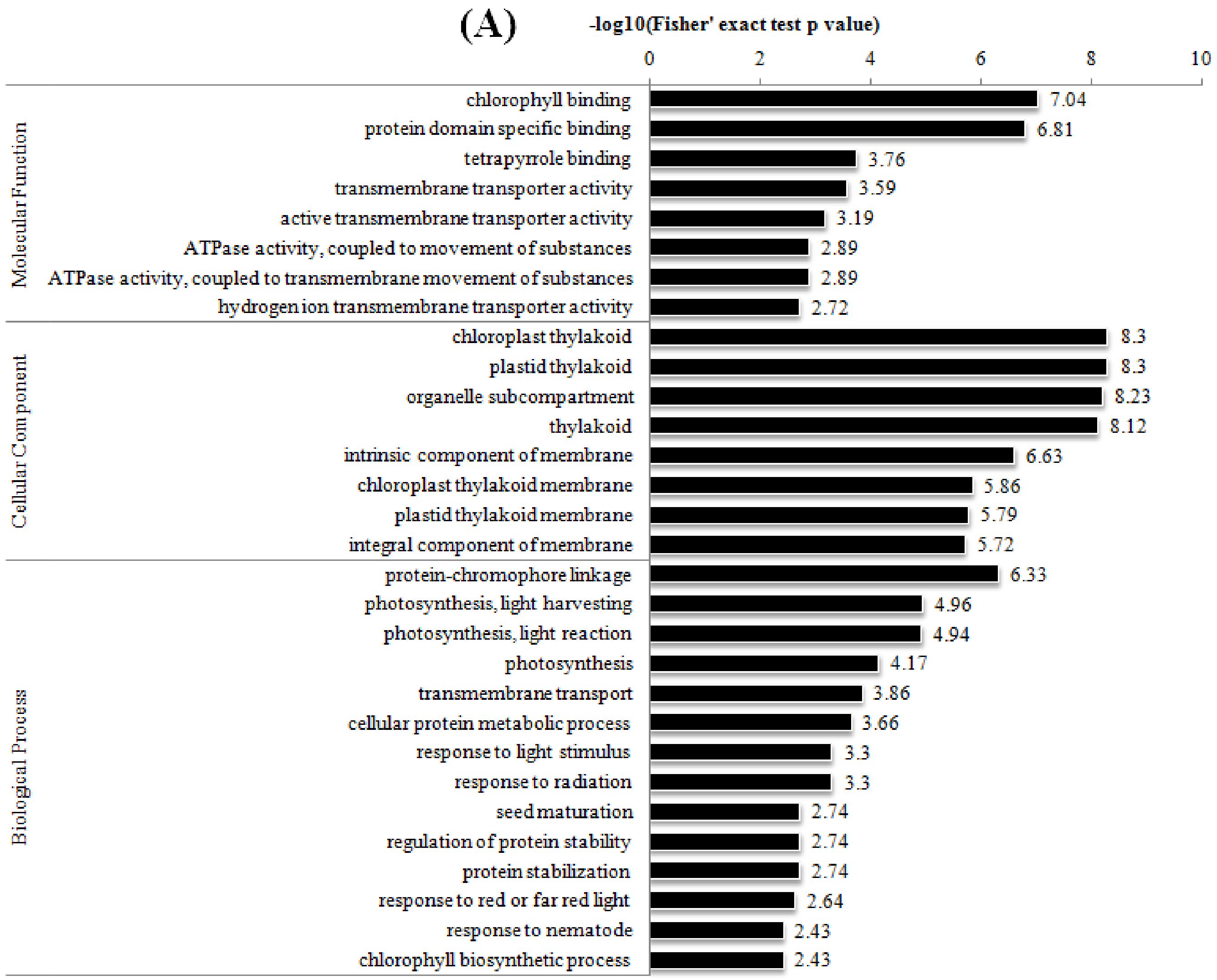
(A)
-log10(Fisher' exact test p value)
0
2
4
6
8
10
Molecular Function
Cellular Component
Biological Process
chlorophyll binding
7.04
protein domain specific binding
6.81
tetrapyrrole binding
3.76
transmembrane transporter activity
3.59
active transmembrane transporter activity
3.19
ATPase activity, coupled to movement of substances
2.89
ATPase activity, coupled to transmembrane movement of substances
2.89
hydrogen ion transmembrane transporter activity
2.72
chloroplast thylakoid
8.3
plastid thylakoid
8.3
organelle subcompartment
8.23
thylakoid
8.12
intrinsic component of membrane
6.63
chloroplast thylakoid membrane
5.86
plastid thylakoid membrane
5.79
integral component of membrane
5.72
protein-chromophore linkage
6.33
photosynthesis, light harvesting
4.96
photosynthesis, light reaction
4.94
photosynthesis
4.17
transmembrane transport
3.86
cellular protein metabolic process
3.66
response to light stimulus
3.3
response to radiation
3.3
seed maturation
2.74
regulation of protein stability
2.74
protein stabilization
2.74
response to red or far red light
2.64
response to nematode
2.43
chlorophyll biosynthetic process
2.43

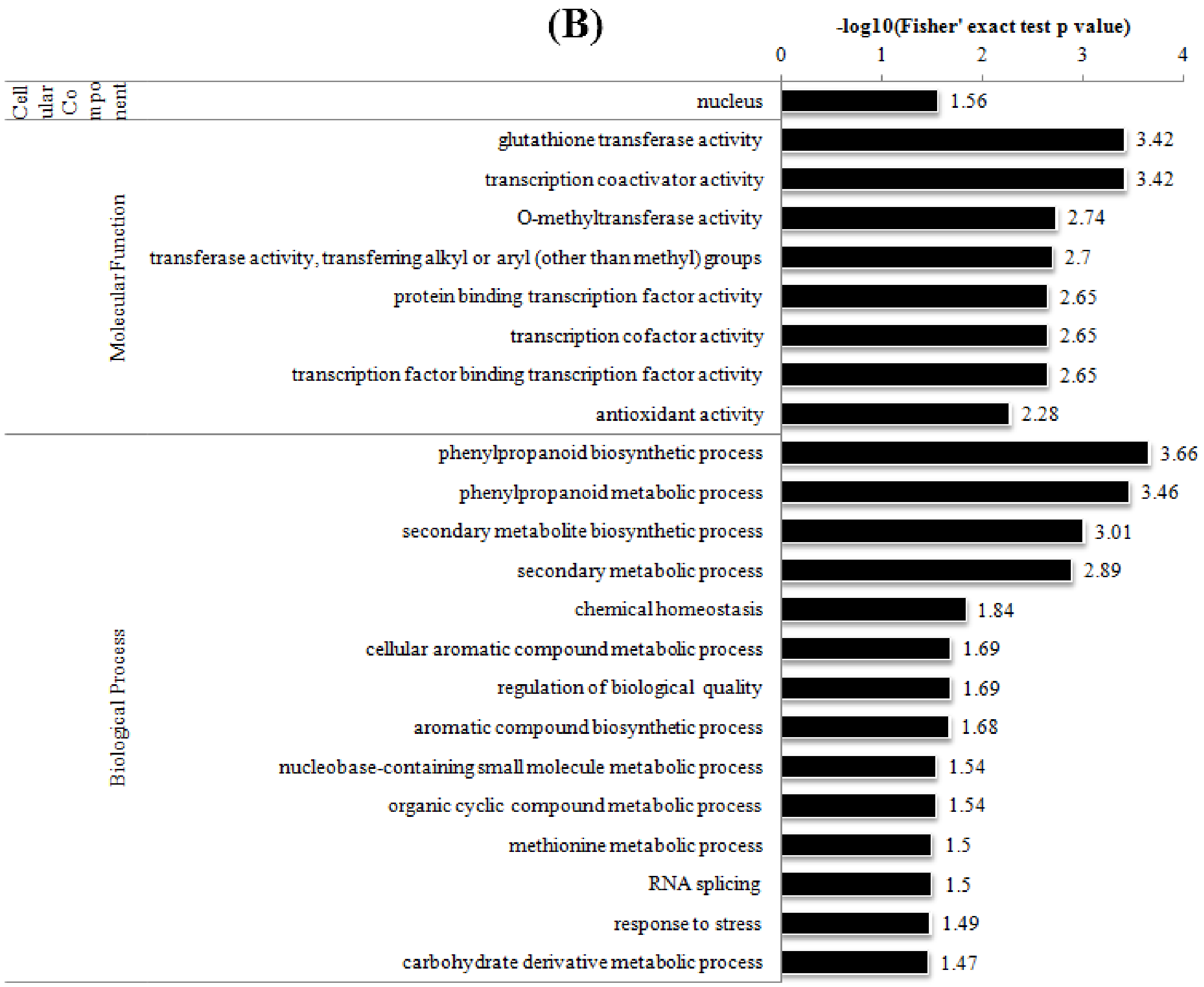
(B)
-log10(Fisher' exact test p value)
0
1
2
3
4
Cellular Component
nucleus
1.56
Molecular Function
glutathione transferase activity
3.42
transcription coactivator activity
3.42
O-methyltransferase activity
2.74
transferase activity, transferring alkyl or aryl (other than methyl) groups
2.7
protein binding transcription factor activity
2.65
transcription cofactor activity
2.65
transcription factor binding transcription factor activity
2.65
antioxidant activity
2.28
Biological Process
phenylpropanoid biosynthetic process
3.66
phenylpropanoid metabolic process
3.46
secondary metabolite biosynthetic process
3.01
secondary metabolic process
2.89
chemical homeostasis
1.84
cellular aromatic compound metabolic process
1.69
regulation of biological quality
1.69
aromatic compound biosynthetic process
1.68
nucleobase-containing small molecule metabolic process
1.54
organic cyclic compound metabolic process
1.54
methionine metabolic process
1.5
RNA splicing
1.5
response to stress
1.49
carbohydrate derivative metabolic process
1.47

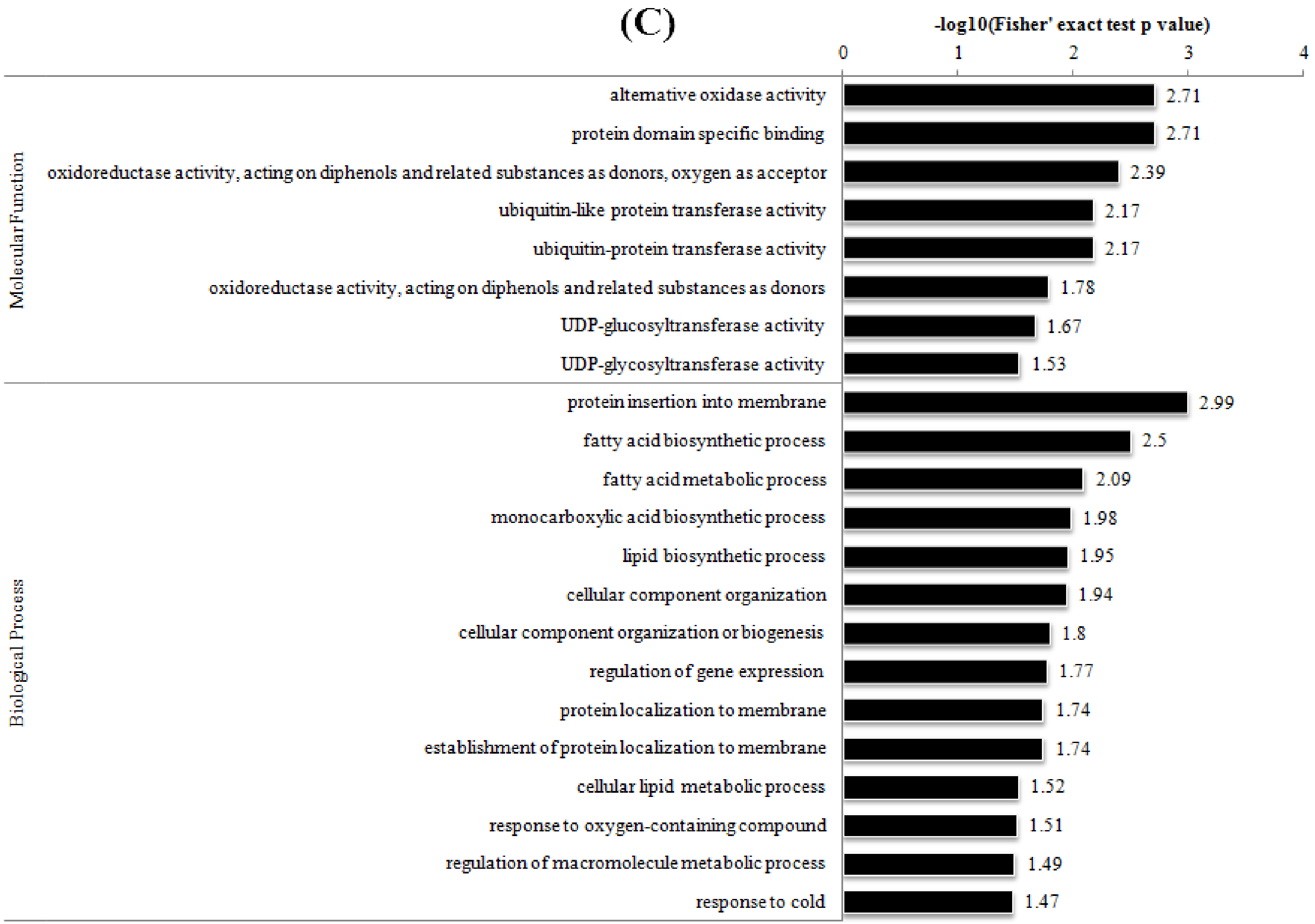
(C)
-log10(Fisher' exact test p value)
0
1
2
3
4
Molecular Function
alternative oxidase activity
2.71
protein domain specific binding
2.71
oxidoreductase activity, acting on diphenols and related substances as donors, oxygen as acceptor
2.39
ubiquitin-like protein transferase activity
2.17
ubiquitin-protein transferase activity
2.17
oxidoreductase activity, acting on diphenols and related substances as donors
1.78
UDP-glucosyltransferase activity
1.67
UDP-glycosyltransferase activity
1.53
Biological Process
protein insertion into membrane
2.99
fatty acid biosynthetic process
2.5
fatty acid metabolic process
2.09
monocarboxylic acid biosynthetic process
1.98
lipid biosynthetic process
1.95
cellular component organization
1.94
cellular component organization or biogenesis
1.8
regulation of gene expression
1.77
protein localization to membrane
1.74
establishment of protein localization to membrane
1.74
cellular lipid metabolic process
1.52
response to oxygen-containing compound
1.51
regulation of macromolecule metabolic process
1.49
response to cold
1.47

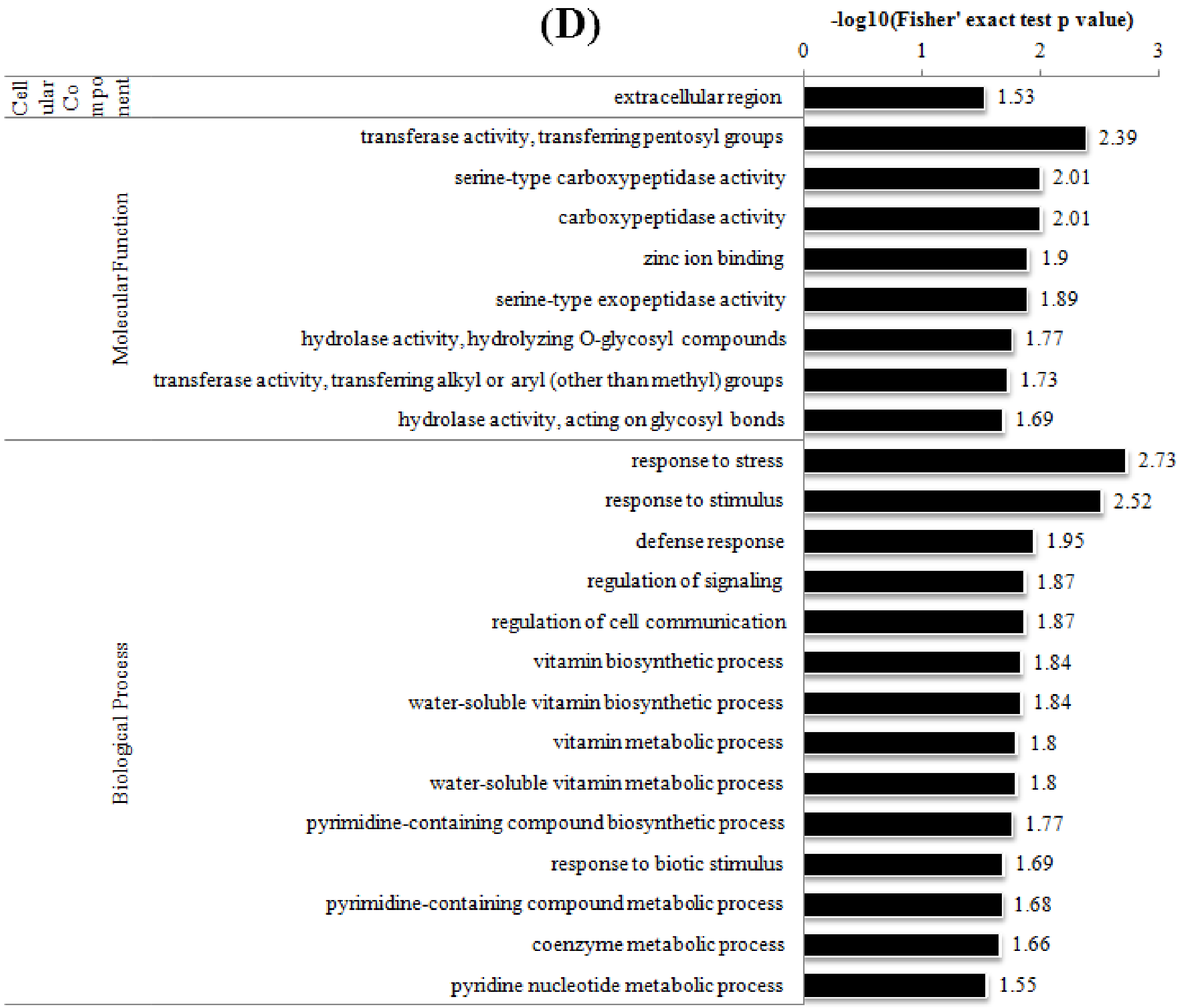

(D)
-log10(Fisher' exact test p value)
0
1
2
3
Cellular Component
Molecular Function
Biological Process
extracellular region
1.53
transferase activity, transferring pentosyl groups
2.39
serine-type carboxypeptidase activity
2.01
carboxypeptidase activity
2.01
zinc ion binding
1.9
serine-type exopeptidase activity
1.89
hydrolase activity, hydrolyzing O-glycosyl compounds
1.77
transferase activity, transferring alkyl or aryl (other than methyl) groups
1.73
hydrolase activity, acting on glycosyl bonds
1.69
response to stress
2.73
response to stimulus
2.52
defense response
1.95
regulation of signaling
1.87
regulation of cell communication
1.87
vitamin biosynthetic process
1.84
water-soluble vitamin biosynthetic process
1.84
vitamin metabolic process
1.8
water-soluble vitamin metabolic process
1.8
pyrimidine-containing compound biosynthetic process
1.77
response to biotic stimulus
1.69
pyrimidine-containing compound metabolic process
1.68
coenzyme metabolic process
1.66
pyridine nucleotide metabolic process
1.55

Figure S2. Gene ontology (GO) classification of differentially accumulated proteins: (A) up-regulated proteins with S_2 treatment compared to S_0 treatment; (B) down-regulated proteins with S_2 treatment compared to S_0 treatment; (C) up-regulated proteins with S_4 treatment compared to S_0 treatment; and (D) down-regulated proteins with S_4 treatment compared to S_0 treatment. S_0 refers to control (no leaf removal); S_2 and S_4 refer to the removal of two and four upper leaves, respectively.
