# Supplementary material for: Physiological and proteome studies of maize (Zea mays L.) in response to leaf removal under high plant density
Source: BMC Plant Biol. 2018 Dec 29;18:378. doi: 10.1186/s12870-018-1607-8 (PMC6310946; doi:10.1186/s12870-018-1607-8)
Supplement: Supplementary file 6 — Figure S3. Effects of leaf removal on relative expression of photosynthesis related proteins (A), the corresponding encoding genes (B) in S2 and relative expression of fatty acid metabolism related proteins (C) and the encoding genes (D) in S4, compared to S0 respectively. The gene candidates are selected by proteins which accumulate in photosynthesis and fatty acid biosynthetic process terms in Fig. 4. S0 refers to no leaf removal (control); S2 and S4 refer to the removal of two or four uppermost leaves, respectively. Data are means ± SE (n = 3). * indicates the significant difference at P ≤ 0.05 level. (PDF 57 kb) [file 12870_2018_1607_MOESM6_ESM.pdf]

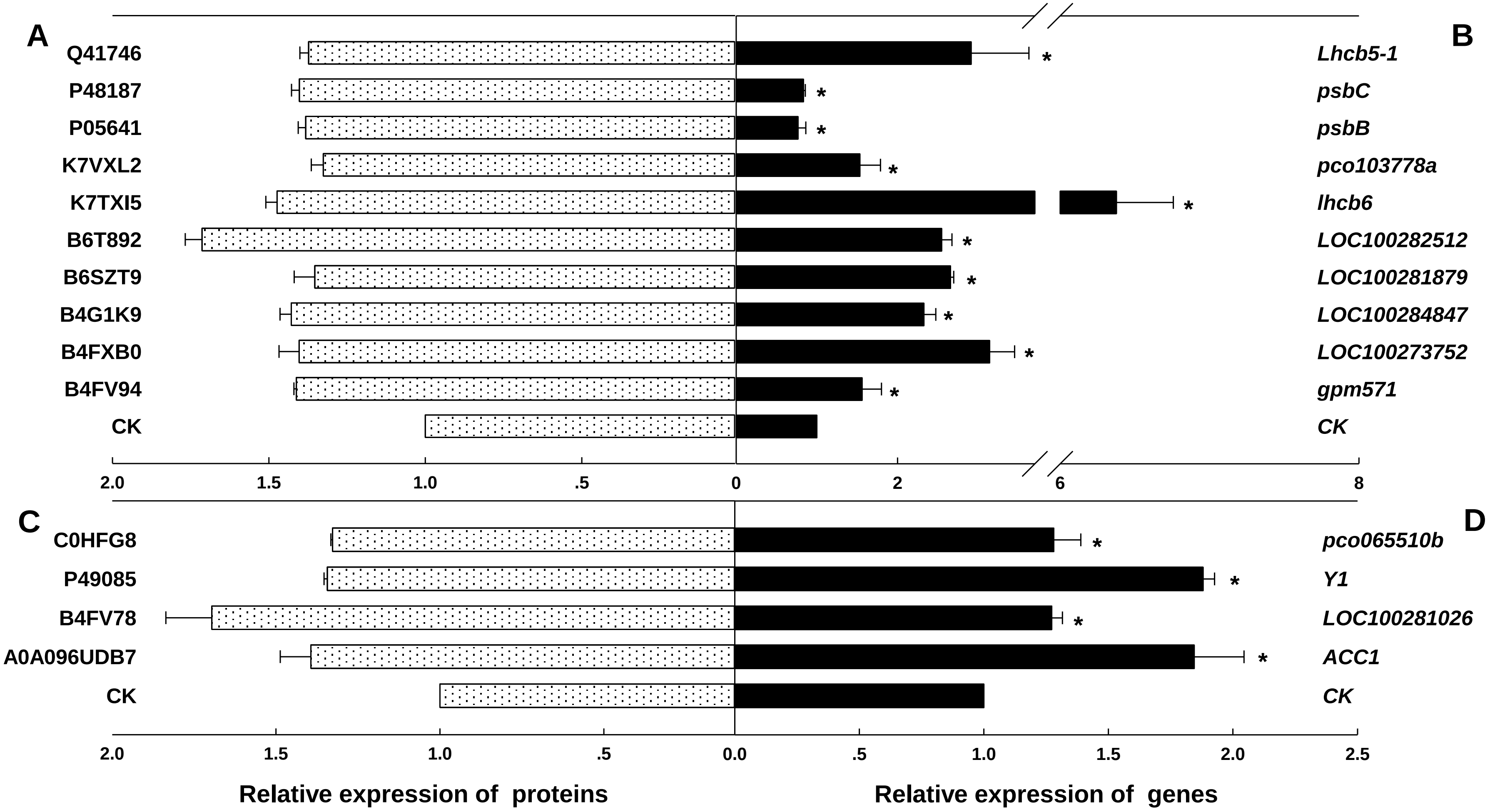

Figure S3. Effects of leaf removal on relative expression of photosynthesis related proteins (A), the corresponding encoding genes (B) in S_2 and relative expression of fatty acid metabolism related proteins (C) and the encoding genes (D) in S_4, compared to S_0 respectively. The gene candidates are selected by proteins which accumulate in photosynthesis and fatty acid biosynthetic process terms in Fig 4. S_0 refers to no leaf removal (control); S_2 and S_4 refer to the removal of two or four uppermost leaves, respectively. Data are means ± SE (n = 3). * indicate the significant difference at $P \leq 0.05$ level compared to S_0.
