# Supplementary material for: Physiological and proteome studies of maize (Zea mays L.) in response to leaf removal under high plant density
Source: BMC Plant Biol. 2018 Dec 29;18:378. doi: 10.1186/s12870-018-1607-8 (PMC6310946; doi:10.1186/s12870-018-1607-8)
Supplement: Supplementary file 7 — Figure S4. Effects of leaf removal on relative expression of defense related proteins (A) and the encoding genes (B) in S2 and S4 compared to S0. S0 refers to no leaf removal (CK); S2 and S4 refer to the removal of two or four leaves, respectively. Data are means ± SE (n = 3). Different lowercase letters indicate the significant difference at P ≤ 0.05 level. (PDF 16 kb) [file 12870_2018_1607_MOESM7_ESM.pdf]

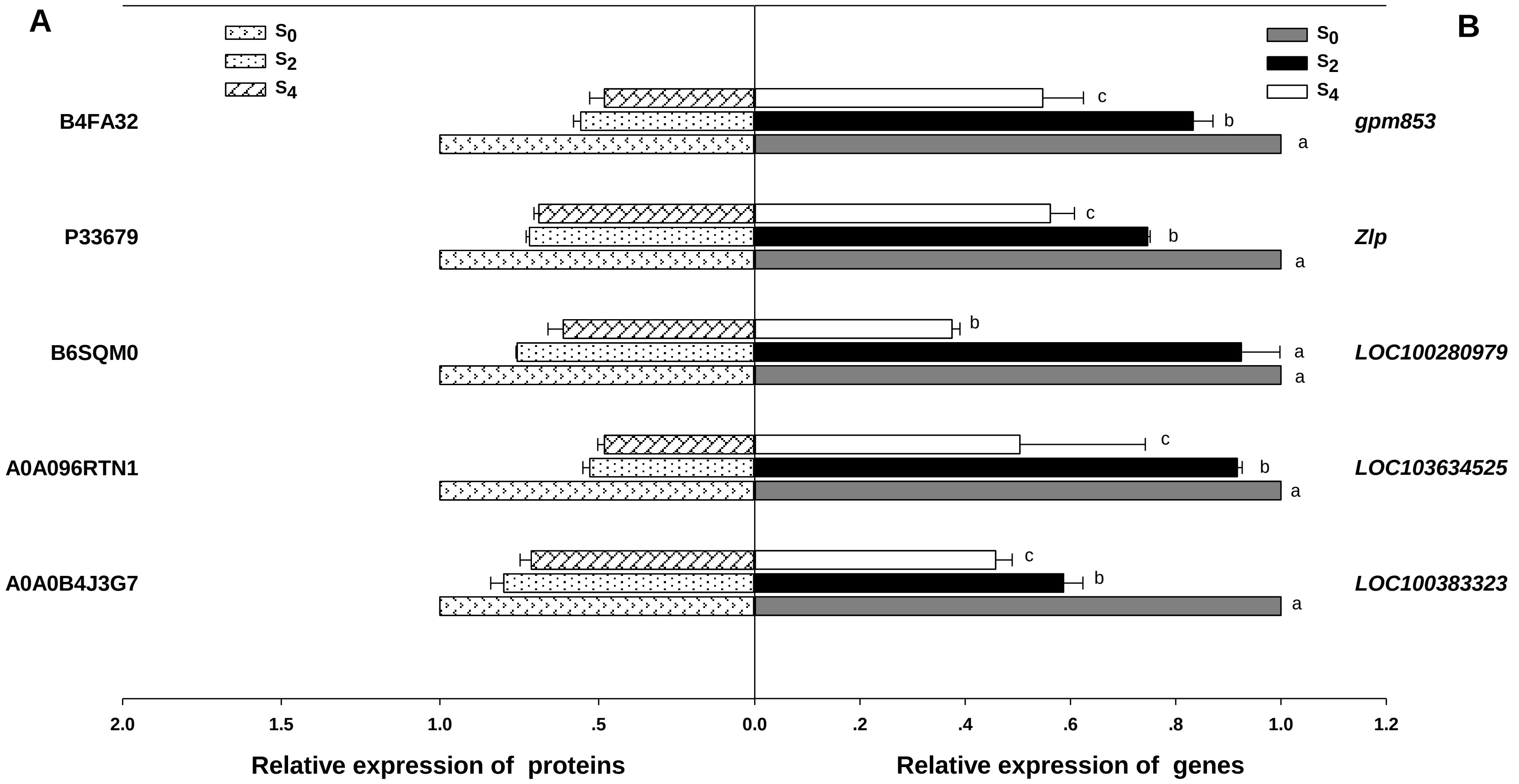

Figure S4. Effects of leaf removal on relative expression of defense related proteins (A) and the encoding genes (B) in S_2 and S_4 compared to S_0. S_0 refers to no leaf removal (CK); S_2 and S_4 refer to the removal of two or four leaves, respectively. Data are means ± SE (n = 3). Different lowercase letters indicate the significant difference at $p < 0.05$ level.
